# CD8ɑ+ cells suppress SIV replication without the development of mutations within MHC class-I-restricted epitopes during post-treatment control

**DOI:** 10.1128/jvi.00041-26

**Published:** 2026-06-15

**Authors:** Ryan V. Moriarty, Olivia E. Harwood, Ethan P. Johnson, William Gardner, Corina C. Valencia, Andrew Conchas, Taina T. Immonen, Matthew R. Reynolds, Brandon F. Keele, Shelby L. O'Connor

**Affiliations:** 1 Department of Pathology and Laboratory Medicine, University of Wisconsin-Madison 5228, Madison, Wisconsin, USA; 2 Microbiology Doctoral Training Program, University of Wisconsin-Madison 5228, Madison, Wisconsin, USA; 3 Wisconsin National Primate Research Center, University of Wisconsin-Madison 5228, Madison, Wisconsin, USA; 4 AIDS and Cancer Virus Program, Frederick National Laboratory for Cancer Research 437329 , Frederick, Maryland, USA; 5 Department of Pathobiological Sciences, University of Wisconsin-Madison 5228, Madison, Wisconsin, USA; University Hospital Tübingen, Tübingen, Germany

**Keywords:** immune escape, viral populations, Mauritian cynomolgus macaque, post-treatment control

## Abstract

Post-treatment control (PTC) is a rare phenomenon in which people living with HIV (PLWH) maintain viral control following antiretroviral treatment (ART) interruption. Characterizing virus populations present in PTCs may help elucidate mechanisms of immunologic control, but this is challenging without detectable plasma viremia. To model PTC, eight Mauritian cynomolgus macaques (MCMs) were infected with barcoded SIVmac239M and began an 8-month ART regimen 2 weeks post-infection (wpi). Six months following ART interruption, all MCMs were rechallenged with non-barcoded SIVmac239, followed by CD8α+ cell depletion 2 months later. Animals were grouped as viremic (*n* = 5) or aviremic (*n* = 3) based on the detection of plasma viremia between ART interruption and CD8ɑ+ cell depletion; all animals became viremic post-depletion. Barcode sequencing of plasma virus revealed that lineages with high pre-ART viral loads dominated the rebounding populations post-depletion and detectable rechallenge virus in two animals post-depletion. Additional sequencing of three CD8+ T-cell epitopes within plasma viruses identified point mutations only in viruses isolated from the viremic group post-depletion. A second cohort of 5 MCM who initiated ART 8 wpi was examined to identify the impact of the timing of ART initiation on viral epitope diversity and showed increased diversity prior to ART initiation and following ART interruption. These results suggest that early ART initiation is associated with reduced diversity within cytotoxic T lymphocyte (CTL) epitopes and a longer time to rebound, as well as highlight an important role of restricting the emergence of variant CTL epitopes in increasing the likelihood of PTC.

**IMPORTANCE:**

While rare, a subset of PLWH, termed post-treatment controllers (PTCs), maintains viral control following antiretroviral treatment (ART) interruption. However, little is known about whether this control reflects a complete absence of viral replication or continual, subclinical replication. Here, we address a key knowledge gap regarding how viral populations change during CD8ɑ+ cell-mediated PTC of SIV. We utilized our Mauritian cynomolgus macaque model of HIV infection, in combination with barcoded SIVmac239M and deep sequencing, to characterize viral lineages and MHC-I-restricted CD8+ T-cell epitopes throughout the study. Our findings demonstrate that early ART initiation limits viral diversity, that pre-ART replication predicts post-ART reactivation, and that CD8ɑ+ cells can suppress viral replication without the emergence of mutations within CD8+ T-cell epitopes. These insights establish MCMs as a valuable model for dissecting mechanisms of durable ART-free viral control and highlight the potential of CD8-mediated immune control as a therapeutic target for HIV cure strategies.

## INTRODUCTION

While antiretroviral treatment (ART) is required for most people living with HIV (PLWH) to maintain viral control, rare individuals, termed post-treatment controllers (PTCs), can maintain undetectable viremia for months to years following ART interruption (1–4). Unfortunately, the low frequency (approximately 4%–16%) of these individuals in the population (3, 5), combined with their low or undetectable viremia, has hindered comprehensive analyses of viral population diversity, replication dynamics, and reservoir stability between PTCs and ART-treated individuals. Studies of durably suppressed, ART-treated individuals report virtually no evidence of viral evolution in proviral DNA sequences sampled during treatment or in plasma viruses isolated at the time of ART interruption (6–9), suggesting efficient inhibition of viral replication. When examining the sequence of replication-competent virus during transient viremia or following ART interruption, the most commonly identified viruses in the rebounding population were typically those present at the highest frequency at the time of therapy initiation (10–13), suggesting little change in the composition of the reservoir during ART. Due to the limited data available for PTCs, it is not known whether the composition of the replication-competent viral reservoir is stagnant during the time when HIV is controlled after stopping ART, or if HIV is replicating at levels below the limit of detection, leading to increased within-host viral diversity and the potential emergence of immune escape mutations.

Cytotoxic CD8+ T lymphocytes (CTLs) play a critical role in the control of HIV/Simian immunodeficiency virus (SIV) infection and are a major driver of viral evolution (14, 15). High frequencies of potent, virus-specific CTL responses have been associated with post-peak viral load decline and viral suppression during acute infection (16, 17). In response to this CTL-mediated selective pressure, immune escape variants within MHC-I restricted CD8+ T-cell epitopes can emerge (14, 17–19). Although the emergence of CTL escape variants has been associated with loss of viral control (20, 21), CTL escape variants have also been identified in individuals that naturally maintain control of viremia or subsequently re-control viremia following transient loss of control (18, 22, 23). However, it is not understood how PTCs may differ from spontaneous control of untreated viremia and if mutations accumulate within CTL epitopes of viruses found in PTCs during these periods of viral control in the absence of continual ART.

SIVmac239M, a molecularly barcoded, clonal SIV strain, has emerged as a vital tool to examine distinct SIV lineages experimentally. SIVmac239M differs from the clonal SIVmac239 by a 34 bp insertion between *vpx* and *vpr* that allows for the distinction of approximately 10,000 unique viral lineages using next-generation sequencing (24). This unique viral strain has been used to examine the number and identity of rebounding lineages following ART interruption (24, 25), viral reactivation rates (26–29), identify the number of lineages participating in CTL escape (30), and the impact of route of infection on the number of viral lineages present during acute infection (31). Using this molecularly barcoded viral strain, we can examine the persistence of distinct SIVmac239M lineages within an animal over time and how the composition of replication-competent viral lineages within the viral reservoir may change during PTC.

We recently reported that Mauritian cynomolgus macaques (MCMs) that began ART at 2 weeks post-SIVmac239M infection could maintain control of viremia for at least 6 months after ART interruption (32, 33). The animals examined here were previously enrolled in a therapeutic vaccine study involving a heterologous prime-boost-boost strategy during ART, followed by three doses of N803 administered 2 weeks apart upon ART cessation. In that study, the phenotype and function of infection- and vaccine-elicited immune responses were evaluated, along with virologic outcomes including plasma viral load and time to viral rebound. While no significant differences were identified in the original study between vaccine and control groups with respect to viral loads or time to rebound, and antigen-specific immune responses were transient (32), the prior interventions remain an important aspect of the experimental context and potential limitation when interpreting viral population dynamics presented here.

Here, we sought to expand upon the previous study and characterize the viral populations present by determining the number and composition of unique lineages persisting over time and assessing how these factors may influence the likelihood of establishing PTC in these SIVmac239M-infected animals. We utilized deep sequencing to characterize SIV lineages and MHC class I-restricted CD8+ T-cell epitope sequences in plasma viruses isolated from MCM infected with SIVmac239M, treated with an 8-month ART regimen beginning 2 weeks post-infection (wpi), rechallenged with the isogenic, non-barcoded SIVmac239 6 months after ART interruption, and administered a CD8ɑ+ cell-depleting antibody 8 weeks after rechallenge. By sequencing the molecular barcode longitudinally, we quantified the number of unique lineages that established persistent infection, assessed whether isogenic rechallenge or CD8ɑ+ cell depletion impacted the composition of viral lineages in the plasma, and examined susceptibility to isogenic rechallenge.

To further evaluate the effects of early versus late ART initiation on the composition of the plasma viral population, we included a second cohort of MCM infected with SIVmac239 who initiated ART at 8 wpi. We compared the sequence diversity of three MHC class I-restricted viral epitopes in viruses isolated from the two cohorts just prior to ART initiation and when SIV became detectable after stopping ART. Together, our study suggests that, in MCM, earlier ART initiation and CD8ɑ+ cell-mediated viral suppression may restrict viral diversity and increase the likelihood of establishing and maintaining PTC, as well as support the utility of MCMs to evaluate potential mechanisms underlying PTC and important species- and context-specific considerations.

## RESULTS

### Description of the early ART study from which the samples were derived

**Fig 1:**
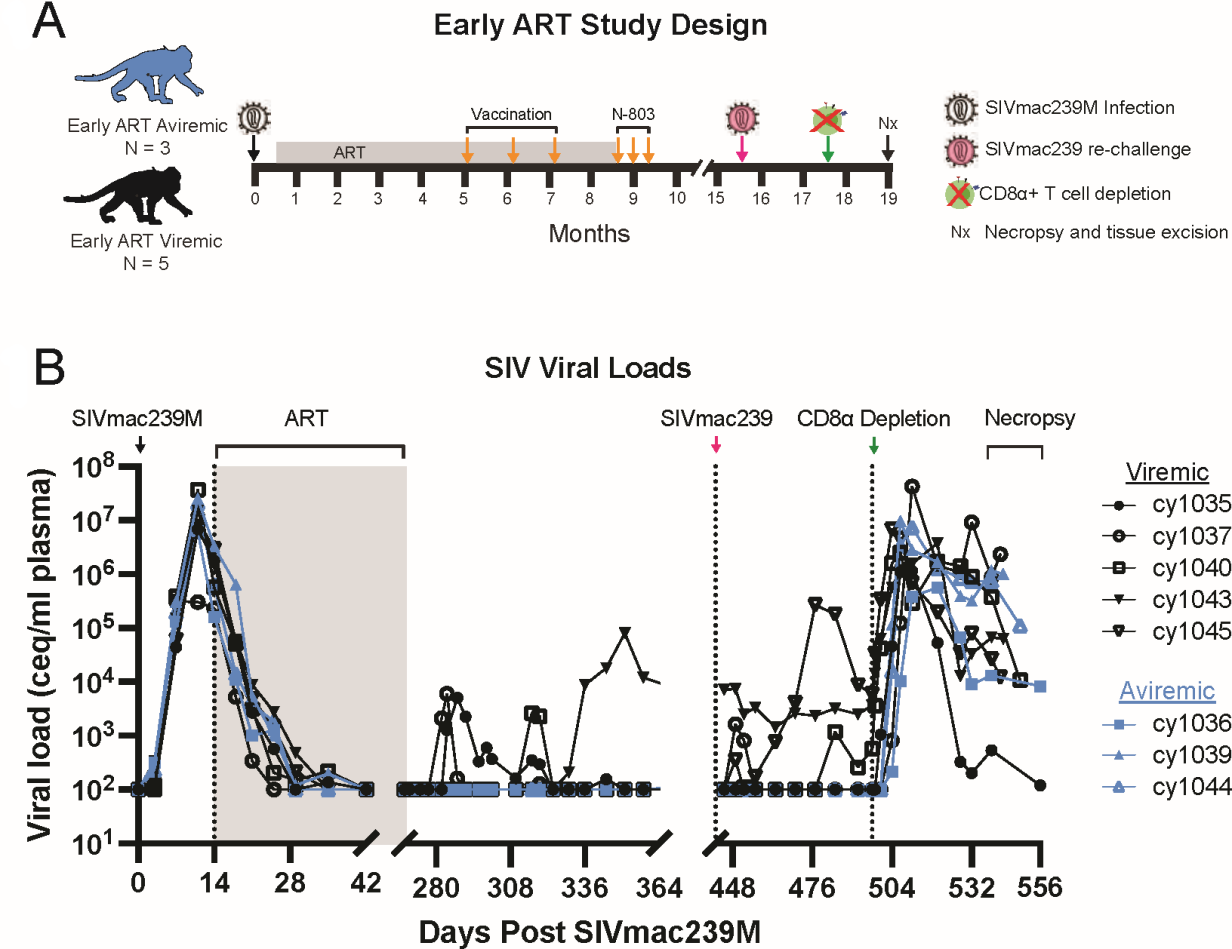
(**A**) Study design and timeline. Intravenous SIVmac239M infection is indicated by the white virion, ART administration is indicated by the gray bar, intravenous SIVmac239 rechallenge is indicated by the pink virion, CD8ɑ+ cell depletion is indicated by the green x-ed out cell, therapeutic interventions are indicated with yellow arrows, and necropsy is indicated by “Nx.” During the ART regimen, four animals were sequentially immunized using recombinant heterologous viral vectors vesicular stomatitis virus (VSV), modified vaccinia virus Ankara strain (MVA), and recombinant adenovirus serotype 5 (rAd-5), all of which expressed SIVmac239 Gag. The vaccinated animals were subsequently treated with three doses of N-803, each 2 weeks apart, beginning on day 3 post-ART. A comprehensive immunologic analysis of this phase is described in Harwood et al. (32). Animals with no detectable viremia between ART interruption and CD8ɑ+ cell depletion are referred to as “Early ART aviremic” (*n* = 3, blue). The remaining animals with detectable viremia above 10^3^ copies/mL of plasma for at least two consecutive time points between ART interruption and CD8ɑ+ cell depletion are referred to as the “Early ART viremic” (*n* = 5, black). (**B**) SIV viral loads over the course of the animal study, with each animal indicated by a unique color and symbol combination, with blue representing aviremic animals and black representing viremic animals. ART was administered between days 14 and 268 post-SIVmac239M infection and is indicated by the gray-shaded area. An intravenous SIVmac239 rechallenge occurred on day 442 post-SIVmac239M infection. Administration of a CD8ɑ+ cell-depleting antibody occurred on day 497 post-SIVmac239M infection. Necropsies were conducted between days 539 and 556 post-SIVmac239M infection. Animals that received immunizations and N803 treatment are shown with closed symbols.

The study timeline and plasma viral loads for each animal are depicted in Fig. 1A and B, respectively. The animals used in this study were previously enrolled in a therapeutic vaccine study described extensively in Harwood et al. (32, 33). Briefly, eight adult male MCM lacking the M1 MHC haplotype associated with spontaneous SIV control (34) were infected intravenously with 10,000 infectious units (IU) of SIVmac239M with daily ART administration consisting of dolutegravir, tenofovir disoproxil fumarate, and emtricitabine beginning 14 days post-infection (dpi). Plasma viral loads peaked between 11 and 14 dpi, and were rapidly suppressed following ART initiation, with no detectable viremia during the 8-month ART regimen.

During ART, four animals (closed symbols) received a heterologous prime-boost-boost therapeutic vaccine regimen of three recombinant viral vectors expressing SIVmac239 Gag. Following ART cessation, vaccinated animals were given three doses of the immunotherapeutic agent N803 administered at 2-week intervals. The remaining four animals received no interventions (open symbols). During the treatment phase, the phenotype and frequency of vaccine-elicited cells and SIV-elicited cells were evaluated (32). While the vaccinated animals mounted greater antigen-specific T-cell responses to both Gag_386–394_GW9 and Gag Pool peptides (32), these differences were no longer significant when the animals were reorganized based on whether they would later become PTCs. Due to the emerging evidence of neutralizing antibodies in establishing and maintaining PTC (35–37), we additionally examined the concentration of bulk anti-SIVmac239 gp120 IgG antibodies in the plasma during and following ART treatment; no differences were identified when animals were grouped by vaccine or control status (33).

Following ART cessation, all animals were monitored for viral rebound for 7 months. During this period, four of eight animals had detectable, often transient, viremia (Fig. 1B, black). However, no association was found between vaccine regimen, post-ART viral loads, and time to viral rebound (32). Additionally, no significant differences in T-cell responses to Gag peptides at either 1 week after the final dose of N803 (4 weeks after ART withdrawal) or 2 weeks prior to rechallenge were observed by IFNγ-ELISPOT when animals were grouped by the presence or absence of detectable viremia (Fig. S1).

All animals were subsequently rechallenged intravenously with 100 TCID_50_ non-barcoded SIVmac239 approximately 7 months following ART cessation, after which one additional animal became viremic. Two months following isogenic rechallenge, a CD8ɑ+ cell-depleting antibody was administered to all animals, resulting in the depletion of both CD8ɑ+ T cells and NK cells and high plasma viremia (33). All animals were necropsied approximately 2 months after depletion.

The animals with no detectable viremia between ART interruption and CD8ɑ+ depletion are hereafter referred to as “aviremic” (*n* = 3; Fig. 1B, blue). The remaining animals with detectable viremia above 10^3^ copies/mL of plasma for at least two consecutive time points between ART interruption and CD8ɑ+ depletion are referred to as the “viremic” cohort (*n* = 5; Fig. 1B, black).

### The number of viral lineages during acute infection was not associated with the detection of transient plasma viremia following ART interruption

**Fig 2:**
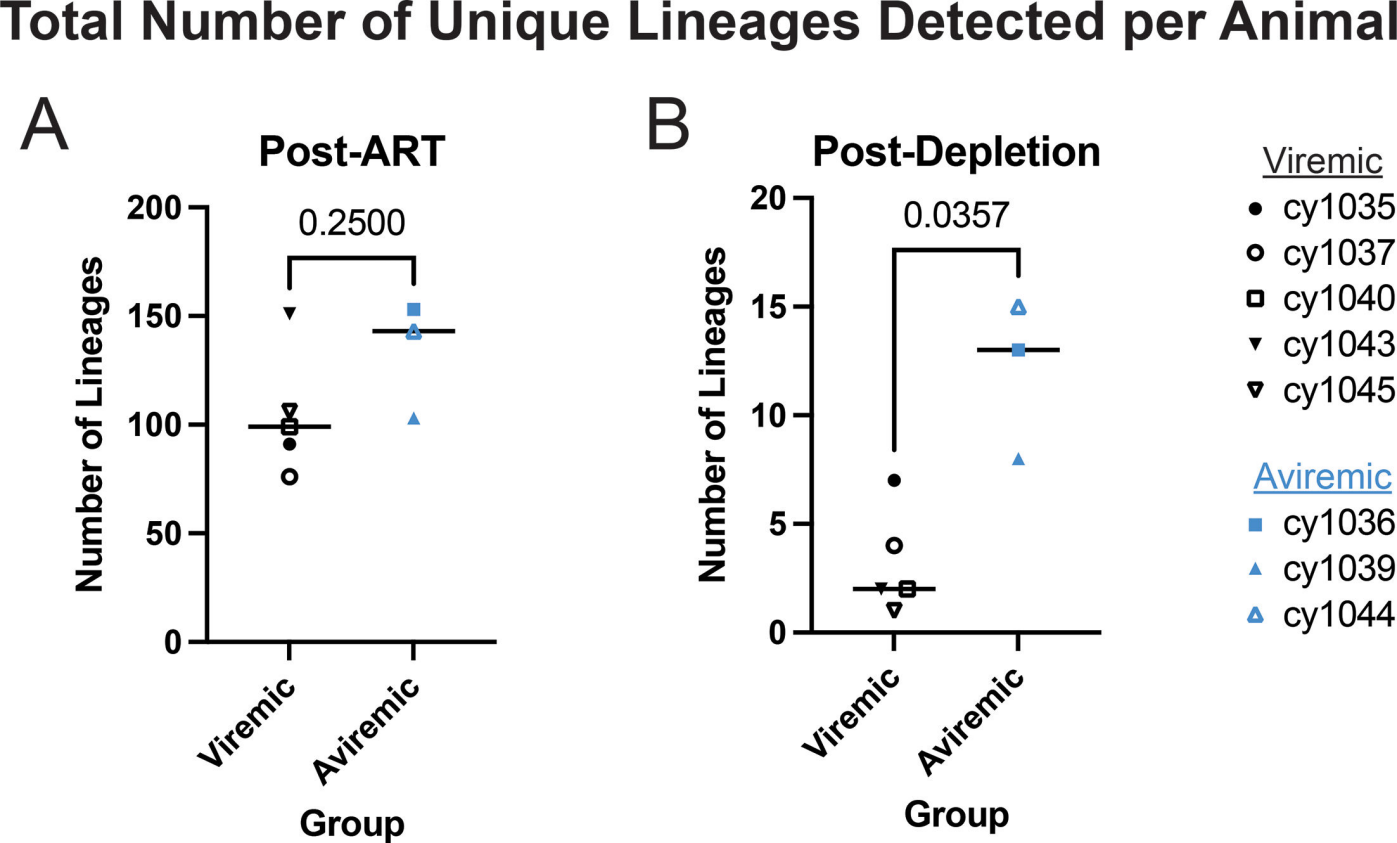
Number of unique SIVmac239M lineages detected in the plasma during the first 2 weeks of SIVmac239M infection, prior to ART initiation (**A**) and following CD8ɑ+ cell depletion (**B**). Animals are coded by unique color and symbol combinations, with blue representing aviremic animals and black representing viremic animals. Only barcodes present at a frequency of 0.002 or greater, which corresponds to 1/minimum input templates of time points pre-ART and post-depletion, were included. Significance is determined by the Mann-Whitney U test.

We first evaluated whether the number of SIVmac239M lineages detectable in the plasma prior to ART initiation was associated with whether an animal rebounded after ART interruption. We sequenced the molecularly barcoded SIVmac239M isolated from plasma and evaluated the total number of unique lineages present in each animal prior to ART initiation and following CD8ɑ+ cell depletion (Fig. 2). We found no difference in the number of detectable lineages at peak, pre-ART plasma between MCMs that rebounded off-ART and those that did not. However, the aviremic animals had a significantly greater number of distinct lineages when compared to the viremic animals following CD8ɑ+ cell depletion (*P* = 0.0357, Mann-Whitney test). These results suggest that while a similar number of lineages may be present during the first 2 weeks following intravenous challenge, there may be an association with viral control following ART interruption and the number of viral lineages that are able to rebound following CD8ɑ+ cell depletion.

### Few viral lineages reactivate following ART interruption

After approximately 8 months of daily ART administration, ART was interrupted, and plasma was monitored for 7 months to detect viral rebound. Three of eight animals (cy1035, cy1037, and cy1040) had transiently detectable viremia and one additional animal (cy1043) had a delayed rebound that was sustained for the rest of the study (Fig. 1B). To evaluate the composition of viral lineages present in the rebounding viral populations, we sequenced the rebounding virus with viral loads greater than 10^3^ copies/mL of plasma in these four animals. We identified between two and four unique lineages from each time point in each animal (Fig. S2A through D), highlighting the strong genetic bottleneck between primary infection (pre-ART) and rebound following ART interruption.

### Most animals were not susceptible to rechallenge with SIVmac239

**Fig 3:**
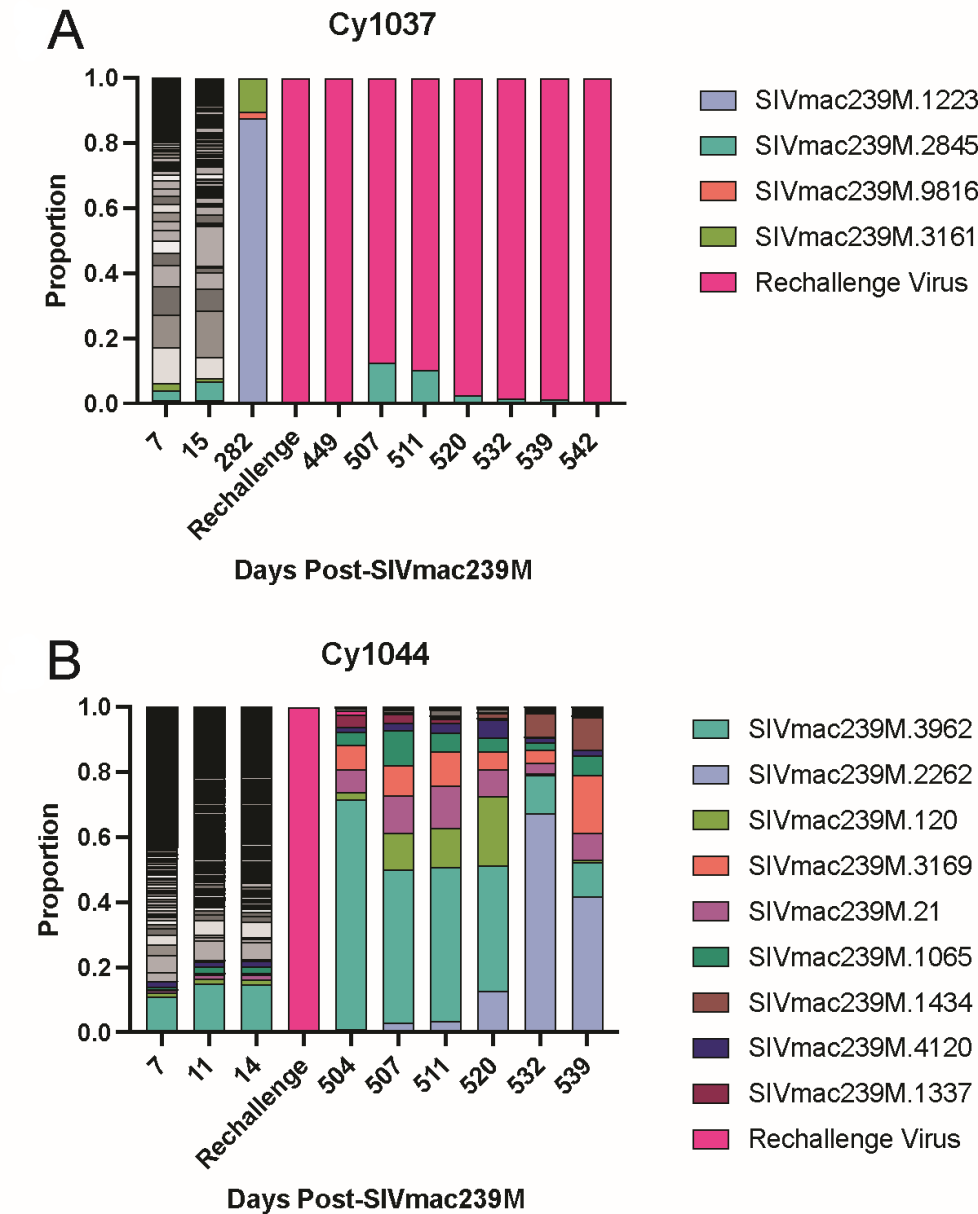
Distribution of SIVmac239M lineages throughout time for the viremic (**A**, CY1037) and aviremic (**B**, CY1044) animals, which had detectable rechallenge virus following intravenous rechallenge. Each unique lineage detected post-ART or post-depletion is indicated by color, with the rechallenge virus indicated in bright pink. In the case of cy1044 (B), only the top 10 lineages detected post-depletion are shown due to color and space constraints; the remaining lineages are shown in shades of gray.

We next determined whether these animals were susceptible to infection with a homologous challenge virus. Approximately 6 months following ART interruption, all animals were intravenously rechallenged with 100 TCID_50_ of the non-barcoded SIVmac239. Rechallenge virus, as identified by the detection of SIV lacking a barcode between *vpx* and *vpr*, was detectable in the plasma immediately following challenge in all animals at a frequency between 94% and 100% (Fig. S3), confirming that the rechallenge virus was successfully administered to each animal. To determine whether the rechallenge virus successfully infected cells and then contributed to the circulating viral population, we sequenced the molecular barcode region of plasma viruses from time points with a viral load of 10^3^ copies/mL of plasma or greater from the day after rechallenge until necropsy (Fig. S2A through H). Interestingly, the rechallenge virus was only detected in one animal (cy1037) immediately following challenge and one additional animal (cy1044) following CD8ɑ+ cell depletion (Fig. 3; Fig. S2B and H). In the case of cy1037, we found that the majority of the detectable virus population post-rechallenge and post-depletion consisted of the rechallenge virus, with only a small proportion (up to 12%) consisting of one other lineage (Fig. 3A; Fig. S2B). In the case of the aviremic animal, cy1044, the rechallenge virus was found only at one time point post-rechallenge, approximately 7 days post-CD8ɑ+ cell depletion at a frequency of approximately 1% (Fig. 3B; Fig. S2H), suggesting that while the rechallenge virus was able to establish infection and contribute to the post-depletion viral population in these two animals, this was limited, replication was transient, and the lineage could be spontaneously controlled.

### Viral lineages detected off-ART typically originate from the highest-replicating lineages pre-ART

**Fig 4:**
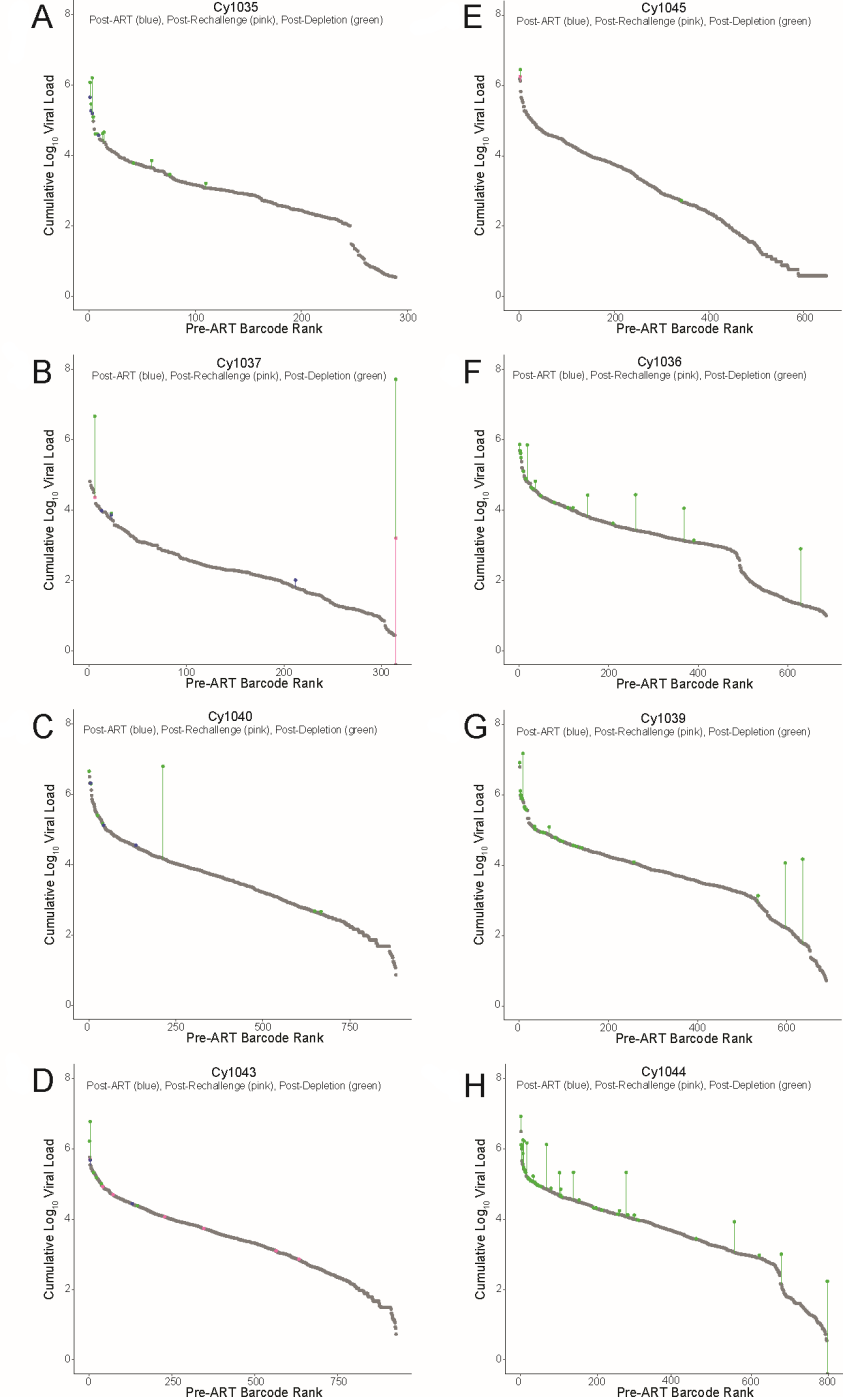
Detection of SIVmac239M lineages following ART interruption (blue), SIVmac239 rechallenge (pink), and after CD8ɑ+ cell depletion (green). Gray dots represent the peak pre-ART viral load of each lineage, with lineages ranked from highest pre-ART replication to lowest pre-ART replication. Panels **A-E** represent viremic animals, and panels **F-H** represent aviremic animals. The rechallenge virus is present on the far right of graphs B (cy1037) and H (cy1044), as this lineage was absent prior to ART initiation but was detected post-rechallenge and depletion.

We next compared the viral lineages detected pre-ART to all the lineages detected after ART interruption (including after CD8ɑ+ cell depletion). We hypothesized that lineages that reactivated following ART interruption would have had the greatest extent of viral replication pre-ART, similar to what was seen previously (25). Each lineage was therefore ranked by viral load at peak viremia (between days 11 and 14 post-infection), with the rechallenge virus last when present. Viral load for each lineage during peak pre-ART viremia is shown in gray and represents the baseline for each barcode lineage, whereas cumulative viral loads for each lineage during each study phase are shown in different colors stemming from the initial peak pre-ART symbol (Fig. 4).

When examining the barcode sequences of the viremic animals, we found that viral lineages detected in rebounding viral populations typically originated from the top of the pre-ART distribution, with lineages replicating to a great extent in pre-ART (gray), post-ART (blue), and post-rechallenge (pink) also replicating the post-depletion (green) population. However, we found that not all lineages present in the post-depletion population also reactivated post-ART or post-rechallenge, such as in animals cy1037 (Fig. 4B) and cy1040 (Fig. 4C). These results suggest that rebound virus can originate from multiple, distinct reservoirs, and that prior replication during a treatment interruption is not required for, or necessarily predictive of, viral replication post-depletion.

Following CD8ɑ+ cell depletion, all animals became viremic. We found that the rebounding lineages post-depletion in previously aviremic animals were distributed widely throughout the peak pre-ART barcode distribution, whereas post-depletion rebounding lineages in viremic animals were predominantly present at the top of the barcode distribution (green symbols, Fig. 4F–H). This result is consistent with previous studies conducted in rhesus macaques who have undergone analytical treatment interruptions both with and without CD8ɑ+ cell depletion (25), supporting the hypothesis that while lineages with high pre-ART viral replication are able to seed a large portion of the viral reservoir and rebound following treatment interruption, even lineages with low pre-ART viral replication contribute to the replication-competent viral reservoir, particularly when CD8-mediated control of viral replication is removed.

### Pre-ART viral replication predicts the likelihood of post-ART and post-depletion lineage reactivation

Following HIV/SIV infection, only a subset of viral lineages establishes productive infection, comprises the replication-competent viral reservoir, and rebounds following treatment interruption (24, 29, 38, 39). However, it is unclear whether the composition of the replication-competent viral reservoir changes during periods of off-ART replication. We hypothesized that the pre-ART viral load area under the curve (AUC), representing its cumulative contribution to the viral reservoir prior to ART initiation, would be a significant predictor of whether that lineage was detected following ART interruption, but prior to isogenic viral rechallenge, suggesting a direct relationship between the size of the lineage-specific viral reservoir and the likelihood of that lineage rebounding post-ART. We further hypothesized that viral replication of a given lineage during periods of transient viremia, either post-ART or post-depletion, would contribute to viral reservoir reseeding and increase the likelihood that the replicating lineage would also be detected following CD8ɑ+ cell depletion.

Consistent with our hypothesis, we found that pre-ART viral load AUC was a significant predictor of reactivation in three of four viremic animals, with animal cy1037 being the exception (Table 1, Model 1, *P* < 0.05). When we extended our analysis to evaluate whether the initial reservoir size was a significant predictor of reactivation following ART until necropsy, we found this was the case in both viremic and aviremic animals, except for cy1037 and cy1045 (Table 1, Model 2, *P* < 0.05).

**TABLE 1:** Summary of logistic regression model parameters^*a*^

			cy1035	cy1037	cy1040	cy1043	cy1045	cy1036	cy1039	cy1044
Post-ART	Model 1: Does pre-ART VL AUC predict post-ART rebound?	AIC	13	34	34	26	–^*b*^	–	–	–
		Intercept (*P*-value)	−36.9 (0.0125)	−7.05 (5.58e-04)	−14.4 (2.33e-04)	−17.4 (1.18e-04)	–	–	–	–
		Pre-ART VL AUC (*P*-value)	6.71 (0.0155)	0.831 (0.111)	1.8 (7.16e-03)	2.36 (0.0298)	–	–	–	–
Post-depletion	Model 2: Does pre-ART VL AUC predict if a lineage will be detected at any point post-ART interruption?	AIC	61	52	103	117	28	161	200	300
		Intercept (p-value)	−15.8 (4.05e-07)	−5.45 (4.88e-07)	−9.46 (4.52e-08)	−11 (2.64e-08)	−9.72 (8.05e-03)	−7 (2.4e-09)	−9.43 (9.79e-12)	−9.84 (2.45e-15)
		Pre-ART VL AUC (p-value)	3.03 (5.35e-06)	0.525 (0.0843)	1.14 (4.66e-04)	1.52 (9.61e-05)	0.977 (0.187)	0.922 (3.88e-04)	1.35 (4.98e-07)	1.55 (2.86e-10)
	Model 3: Does detecting a lineage between ART interruption and isogenic rechallenge improve the likelihood of detecting that lineage post-depletion?	AIC	60	33	86	44	–	–	–	–
		Intercept (*P*-value)	−14 (1.47e-05)	−5.71 (5.66e-06)	−8.63 (2.33e-06)	−22.4 (3e-05)	–	–	–	–
		Pre-depletion VL AUC (*P*-value)	2.62 (2.21e-04)	0.304 (0.434)	0.917 (0.01)	3.45 (2.78e-04)	–	–	–	–
		Post-ART detection (*P*-value)	17.1 (0.993)	3.85 (0.0103)	−13.5 (0.992)	3.44 (0.109)	–	–	–	–
	Model 4: Does detecting a lineage between isogenic rechallenge and CD8ɑ+ cell depletion improve the likelihood of detecting that lineage post-depletion?	AIC	–	17	–	47	21	–	–	–
		Intercept (*P*-value)	–	−11.1 (0.058)	–	−23.2 (2.34e-05)	−6.84 (2.64e-04)	–	–	–
		Pre-depletion VL AUC (*P*-value)	–	1.56 (0.244)	–	3.61 (1.82e-04)	0.128 (0.801)	–	–	–
		Post-rechallenge detection (*P*-value)	–	28.7 (0.994)	–	−13.3 (0.995)	23.6 (0.995)	–	–	–

^*a*^ Model 1 examines the virus population in the plasma following ART interruption but prior to isogenic viral rechallenge (between days 268 and 449 post–SIVmac239M infection). Model 2 examines the plasma virus population at all time points following ART interruption. The sole explanatory variable for Model 1 and Model 2 is the area under the curve (AUC) of a given lineage prior to ART initiation. Models 3 and 4 incorporate both the AUC of a given lineage prior to ART initiation and a binary indicator variable for whether a lineage was detected between ART interruption and isogenic rechallenge (Model 3) or between isogenic rechallenge and CD8ɑ+ cell depletion (Model 4).

^*b*^ –, not applicable.

When evaluating whether replication of a given lineage post-ART (Model 3) or post-rechallenge (Model 4) improved the model, we found that although pre-ART viral load AUC remained a significant predictor for most viremic animals, the inclusion of these variables did not typically improve the model fit, with most comparisons showing similar AIC values (Models 3 and 4 compared to Model 2). Additionally, post-ART or post-rechallenge detection was not a significant predictor of rebound post-depletion in any animal (Models 3 and 4, *P* > 0.1), except for cy1037 (Model 3, *P* = 0.01), whose viral population consisted of primarily the rechallenge virus following isogenic rechallenge (Fig. 3; Fig. S2B). Together, these results suggest minimal influence of post-ART and post-rechallenge detection on the post-depletion population, but do not definitively support or refute the continual reseeding hypothesis (25), likely due to the low level of viral replication that occurred between ART interruption and CD8ɑ+ cell depletion.

CTL epitope sequences present in viruses from the aviremic group matched the inoculum at the time of CD8ɑ+ cell depletion, but accumulated sequence variation as the CD8ɑ+ cells returned

The observed viral rebound associated with depletion of CD8ɑ+ cells indicated that CD8ɑ+ cells played a major role in maintaining PTC (33). To determine whether the existing CD8ɑ+ cells may have contributed to sequence variation during PTC, we characterized the sequences of three MHC class I-restricted CD8+ T-cell epitopes from viruses isolated from seven animals at the time of CD8ɑ+ cell depletion to determine whether they had accumulated variants. Animal cy1037 was excluded from epitope analysis because nearly 100% of the viral population could be attributed to the rechallenge, which precluded the study of viral epitopes in this animal during periods of PTC.

**Fig 5:**
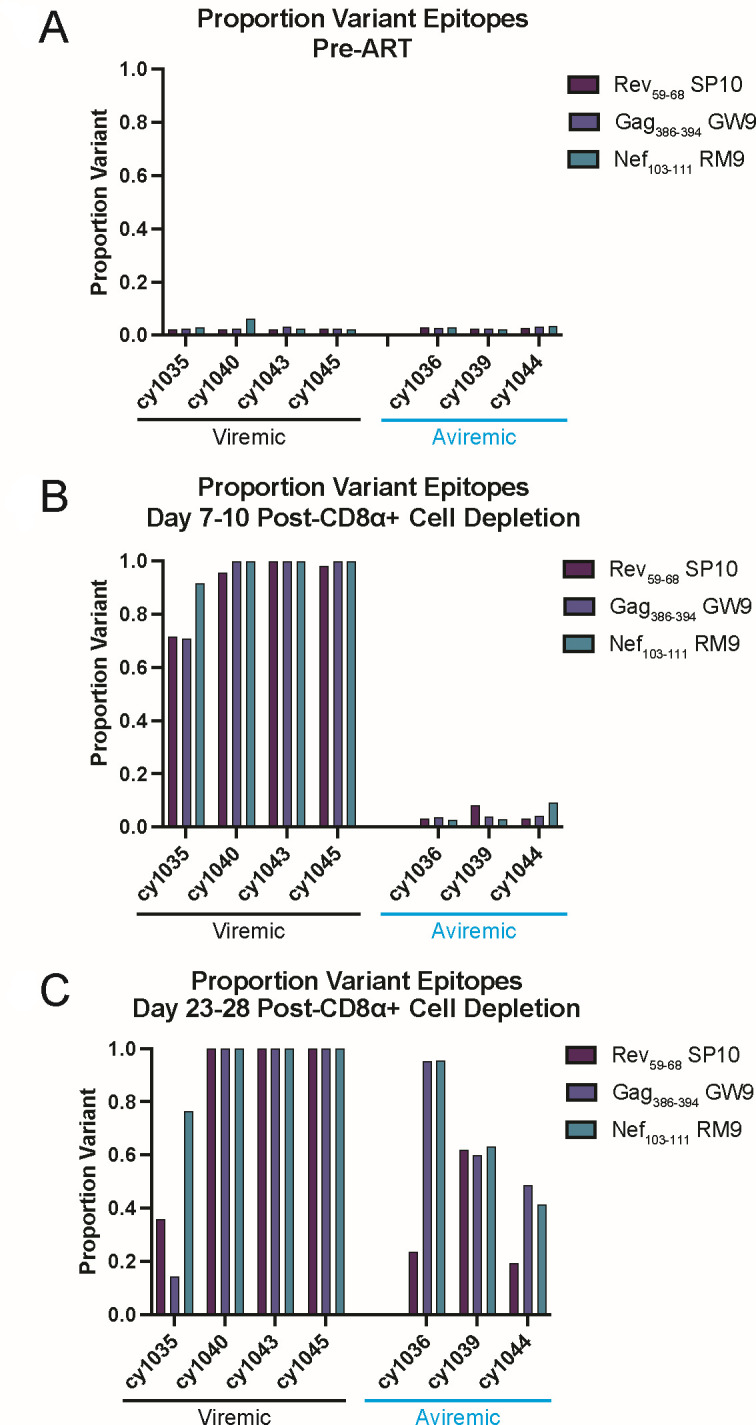
Proportion of variant Gag_386–398_GW9 (blue), Nef_103–111_RM9 (teal), and Rev_59–68_SP10 (purple) epitope sequences in viremic (left, black) and aviremic (right, blue) animals prior to ART initiation (**A**), 7–10 days following CD8ɑ+ cell depletion (**B**), and 21–28 days post-CD8ɑ+ cell depletion (**C**).

All animals expressed at least one copy of the M3 MHC haplotype (Table S1) (32, 33), which contains the Mafa-A1*063 and Mafa-B*075 MHC class I alleles. We focused on two epitopes restricted by Mafa-A1*063 (Gag_386–394_GW9 and Nef_103–111_RM9) and one epitope restricted by Mafa-B*075 (Rev_59–68_SP10) that commonly develop immune escape mutations during acute SIV infection (40). Prior to ART initiation, the CTL epitope sequences were nearly 100% identical to the inoculum, as expected (Fig. 5A). Approximately 1 week after administering the CD8ɑ+ cell-depleting antibody, the three CTL epitope sequences present in viruses from animals in the Early ART aviremic group predominantly matched the inoculum, whereas those present in viruses from the Early ART viremic animals were almost entirely variant (Fig. 5B).

Approximately 3–4 weeks following CD8ɑ+ cell depletion, NK cells and bulk, but not peptide-specific, CD8ɑ+ cells returned in the peripheral blood of most animals (Fig. S4). At this point, viral variants were detectable in the sequences of the examined epitopes from all animals in both groups (Fig. 5C). While we wanted to determine whether unique barcoded SIV lineages emerged coincident with the detection of variant epitopes, the three epitopes we examined were too far away from the barcode (all greater than 2 kb away from the barcode region) to perform a linkage analysis due to the short reads generated by Illumina sequencing.

### Viral rebound in MCMs is associated with the presence of CTL epitope variants present prior to ART initiation

**Fig 6:**
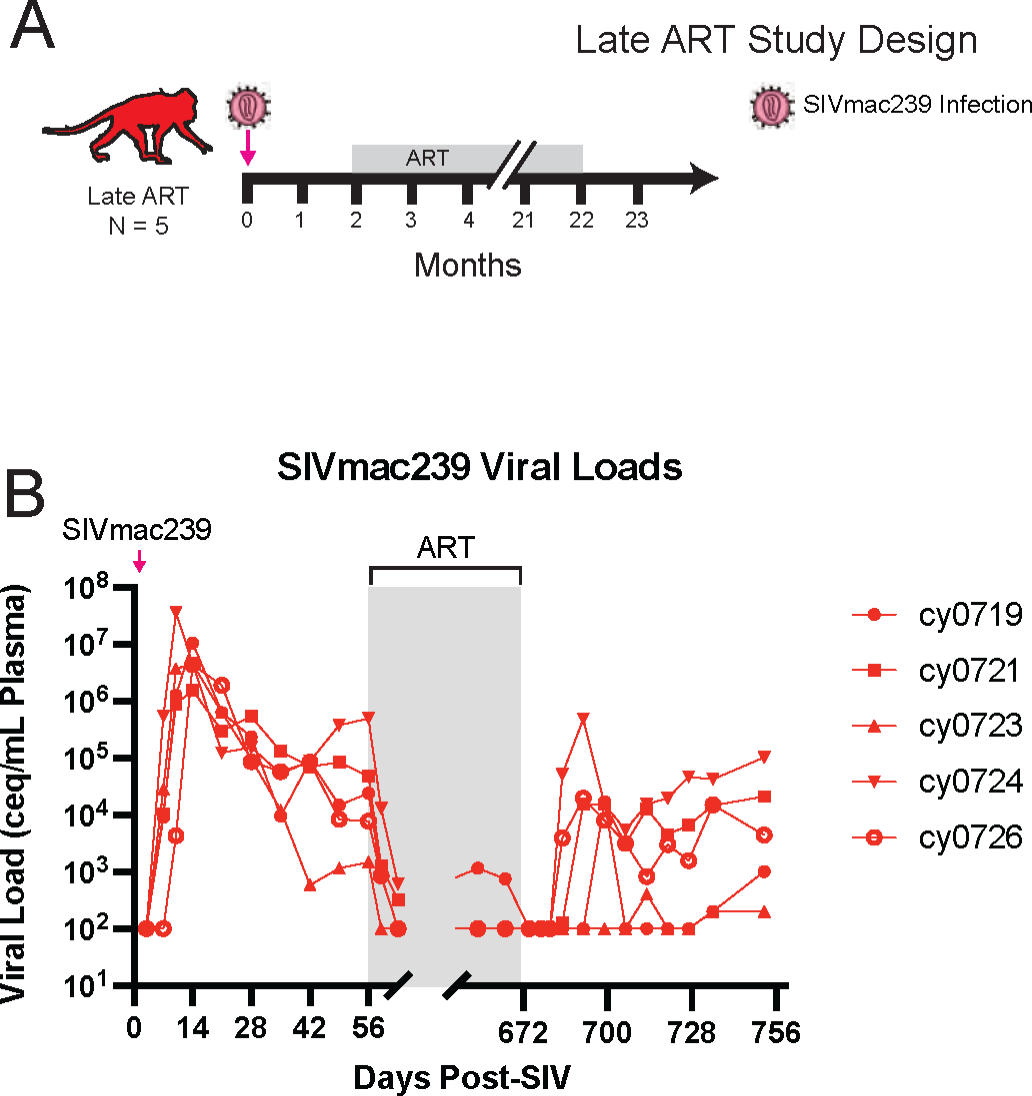
Study design and timeline for the Late ART animals (**A**). All five animals were infected intrarectally with SIVmac239 and began ART 8 weeks post-infection. ART was withdrawn after approximately 22 months. SIV plasma viral loads for the Late ART animals (**B**). ART administration is shown by the gray shaded bar. Animals are indicated by unique symbols.

While the relationship between CTL epitope variation and PTC remains incompletely understood, the presence of sequence variation within CTL epitopes has been associated with poor viral control in untreated HIV/SIV (20, 41, 42). Therefore, we also examined five MCMs described previously (33) that started a 22-month ART regimen 8 weeks post-infection, four of which had at least one copy of the M3 MHC haplotype (Table S1) (33). These animals had detectable, sustained viremia (range 1.25 × 10^2^ to 4.85 × 10^5^ copies/mL) between 2 and 6 weeks after ART interruption (Late ART cohort, Fig. 6) (33), in stark contrast to the Early ART cohort (Fig. 1B).

**Fig 7:**
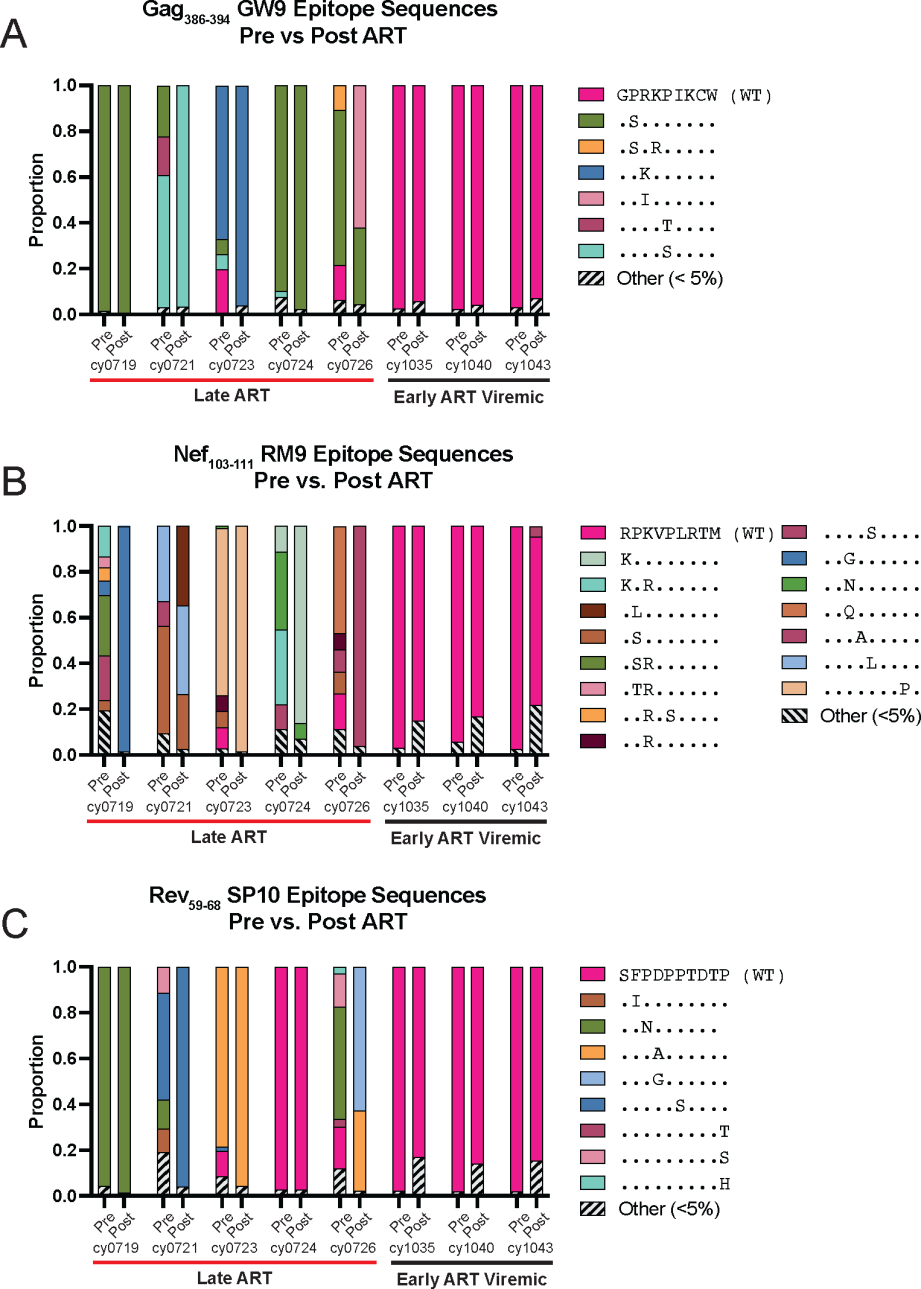
Gag_386–398_GW9 (**A**), Nef_103–111_RM9 (**B**), and Rev_59–68_SP10 (**C**) epitope sequences pre- and post-ART for Late ART (left, red) and Early ART viremic (right, black) animals. The “pre-ART” time point was between 11 and 14 days post-SIVmac239M infection for the Early ART cohort and 49 days post-SIVmac239 for the Late ART cohort. The “post-ART” time point was the first time point following ART interruption with a viral load greater than 10^3^ copies/mL. Wild-type epitope sequences are in pink bars. Epitope sequences that comprised less than 5% of the total population are in the striped gray and black bars. Variant epitope sequences are shown in different colors. Residues matching the wild-type epitope sequences are indicated by dots, and nonsynonymous residues are indicated by letters.

We sequenced the plasma virus from the Late ART cohort and the Early ART viremic animals immediately prior to ART initiation and at the first detection of virus greater than 10^3^ copies/mL of plasma after ART interruption. For each animal, we calculated the composition of variant epitope sequences for the three MHC class I-restricted epitopes Gag_386–394_GW9, Rev_59–68_SP10, and Nef_103–111_RM9. These epitopes were primarily wild type at both time points for the Early ART viremic cohort (Fig. 5A and 7). In contrast, viruses from the Late ART cohort had predominantly variant epitope sequences at both time points, even if the specific variants differed (Fig. 7). To ensure that these differences were not a result of the viral stocks used to infect animals, we evaluated the epitope sequences of the SIVmac239 stock used for the Late ART cohort and the SIVmac239M stock used for the Early ART cohort. The composition of variants within each stock was comparable (Table S5) and consistent with variation present in clonal viral stocks sequenced at high depth (43).

To determine differences in the composition of epitope sequences pre- and post-ART in each group over time, we calculated the Bray-Curtis dissimilarity index, which compares the compositional dissimilarity between two populations, for the three MHC-I-restricted T-cell epitopes examined in each animal. Across all three epitopes, we found that the Late ART cohort had a higher Bray-Curtis value (median >0.37) when compared to the Early ART viremic cohort (median <0.16) (Fig. S5). While statistical significance was not reached, the greater dissimilarity between pre-ART and post-ART time points in the Late ART animals suggests that the presence of mutations within CTL epitopes prior to ART initiation may contribute to continual epitope diversification despite persistent ART treatment and the absence of detectable viremia.

## DISCUSSION

In this study, we took advantage of a unique opportunity to examine viral populations in eight SIVmac239M-infected MCM who initiated ART at 2 weeks post-infection and exhibited variable degrees of PTC, as described in Harwood et al. (32, 33). This allowed us to conclude that while most animals were not susceptible to infection by the rechallenge virus, the two animals that were susceptible differed greatly with respect to the extent of rechallenge virus detectable in the plasma. We also found that lineages rebounding at any point following ART interruption typically had high levels of pre-ART viral replication, and that pre-ART replication, but not post-ART or post-rechallenge replication, was predictive of viral reactivation. Finally, we discovered that the aviremic animals did not develop mutations within MHC class I-restricted CD8+ T-cell epitopes during the period of PTC. Cumulatively, this study highlights the importance of evaluating viral populations in PTCs, specifically with respect to how the composition of these viral populations may be influenced by transient viral replication and selective pressure exerted by host immune responses.

Due to the rarity of PTCs, examination of viral populations within these individuals has been limited and has primarily focused on the size and composition of the proviral reservoir (44, 45). However, understanding if, and to what extent, viral replication and evolution are ongoing in these individuals has major implications for sustained viral control, as the development of immune escape mutations has been found to precede loss of viral control in elite controllers (21, 23). Here, we observed low viral diversity within MHC-restricted CD8+ T-cell epitopes immediately after CD8ɑ+ cell depletion in the aviremic PTCs (Fig. 7), which is consistent with results from human PTCs described recently by Trémeaux et al. (46). Limiting viral replication, and by extention viral evolution, is one goal for HIV therapeutics aimed at treatment-free remission. Future studies that identify the immunological mechanism restricting viral replication among those harboring replication-competent proviruses could identify the essential features of an immunotherapy.

We do not fully understand how transient viremia following ART interruption impacts the composition of the rebound-competent viral reservoir in PTCs. Previous studies conducted in RM showed that while transient viral replication impacted the rebounding population following serial analytical treatment interruptions (ATI), this was limited and required a high degree of viral replication during the ATI (25). We utilized a similar logistic regression approach to determine whether the extent of pre-ART viral replication was a significant predictor of reactivation post-ART and whether reactivation post-ART or post-rechallenge influenced the likelihood of reactivation following depletion (Table 1). Across all animals and models evaluated, pre-ART viral load AUC was the predominant predictor of post-depletion reactivation; detection of a lineage during transient off-ART viremia was a significant predictor of post-depletion reactivation only in animal cy1037, whose viral population consisted primarily of the rechallenge virus. However, we must use caution when interpreting these results due to the low number of unique lineages reactivating during periods of off-ART viremia and the minimal viral replication observed during these periods. As such, while these results suggest that the rebounding viral population will stem from lineages comprising a large portion of the pre-ART population and that this population does not change significantly in the presence of low or undetectable viremia, we cannot definitively state that the reservoir cannot be reseeded or altered following a large burst of viral replication.

Following CD8ɑ+ cell depletion, we observed viral replication in all eight animals (Fig. 1B). Sequencing the viral populations present prior to and following CD8ɑ+ cell depletion allowed us to examine the extent of sequence variation within CTL epitopes during the study (Fig. 5). We found no evidence of immune escape within the three MHC-restricted CD8+ T-cell epitopes evaluated in the aviremic animals at 7–10 days post depletion, but variation was common by ~3–4 weeks post-depletion (Fig. 5). Importantly, given that rechallenge virus was only detected on the day of rechallenge (Fig. S3) and at a single time point in the aviremic animal with detectable rechallenge virus post-depletion (Fig. 3; Fig. S2), the presence of epitope sequences matching the inoculum observed in the plasma is unlikely to be derived from the rechallenge virus.

Detecting wild-type SIV epitope sequences at the first post-depletion time point could be the result of two possibilities. First, there may have been limited viral evolution in the reservoir during PTC in these animals. Alternatively, viruses with wild-type epitope sequences may have had a growth advantage over any variant viruses once the CD8ɑ+ cells were depleted. While we cannot distinguish between these possibilities with this data set, our results are in line with the hypothesis that early ART initiation and the presence of a small viral reservoir in MCM preserve wild-type epitope sequences such that the CTL are more capable of virus suppression at the time of ART interruption. Based on the 3- to 4-week delay in variant detection observed here, we posit that SIV did not evolve during PTC in the aviremic animals because CD8+ cells were acting to suppress viral replication. One alternative hypothesis for the absence of immune escape variants despite CD8+ cell-mediated control is a non-cytotoxic CD8+ T-cell mechanism that acts to suppress virus transcription without selecting for CTL escape variants (47–49). Future studies will need to compare viral variation from the reservoir during ART to that which is present after ART interruption in MCMs to characterize the extent to which antiviral CD8+ cells may be able to restrict viral replication and evolution during periods of PTC, potentially by employing next-generation sequencing technologies such as single-cell RNAseq or single-genome amplification of viruses present in tissues.

Initiating ART during early HIV infection remains one of the most highly supported interventions contributing to the increased likelihood of establishing PTC, with additional therapies, including broadly neutralizing antibodies and HIV CAR T cells, under investigation (3, 50–54). We found that SIV-infected MCMs that began ART 8 wpi had a shorter time to viral rebound and a greater extent of pre- and post-ART viral sequence diversity within the CTL epitopes examined when compared to animals that initiated ART at two wpi (Fig. 1B, 6B and 7, and Fig. S5). These results are consistent with previous studies reporting associations between later ART initiation, increased viral diversity, and shorter time to viral rebound following treatment interruption (55, 56), as well as limited viral evolution and the maintenance of immune escape variants during ART treatment (6, 57). Importantly, we determined that animals with later ART initiation had an increased number of viral variants present in the replicating viral population when ART was initiated. While it would be beneficial to examine any potential linkage between unique epitope variants and distinct viral lineages with PTC, we were unable to evaluate this aspect of the viral population, given the short reads generated by Illumina sequencing and the absence of barcoded SIV in the Late ART cohort. Together, our study supports the hypothesis that even a slight delay in ART initiation may allow for new variants to accumulate in the viral population, both prior to ART initiation and during ART treatment, reducing the possibility of establishing PTC. However, because we did not directly evaluate whether these variants impacted functional recognition, these observations must be interpreted as descriptive rather than causative.

Importantly, viruses isolated from the animals in the Late ART cohort had mutations in the CTL epitopes examined prior to ART initiation, which may have impacted the ability of CD8+ T cells to suppress viral replication after ART interruption. However, we were unable to conduct functional assays to determine whether these mutations constituted immune escape due to sample constraints. To better understand the impact of potential immune escape on time to viral rebound following ART interruption, future studies could consider using a pre-escaped challenge virus in MCMs with defined MHC genetics. Pre-escaped viruses have previously been used to show impaired viral control during acute infection when compared to non-escaped, wild-type virus (58), as well as inefficient recognition by CTL (42). When applied to future studies of PTC, the inclusion of similar pre-escaped viruses may allow researchers to evaluate the extent to which antiviral CD8+ T-cell responses are required to establish and maintain PTC, as pre-existing CTL responses to the viral inoculum would be absent.

Unique to this study, we examined whether the PTCs were susceptible to rechallenge with an isogenic viral strain by quantifying the proportion of virus containing a barcode, representing the initial challenge virus (SIVmac239M), and the proportion lacking a barcode, representing the rechallenge virus (SIVmac239). Previous studies have shown that animals infected with an attenuated SIV strain or a Simian-human immunodeficiency virus (SHIV) strain were afforded protection from rechallenge with a similar, but genetically distinct, virus (59–62). Similar to these studies, we found that while most animals were protected from isogenic rechallenge, as determined by the low frequency of detectable rechallenge virus (SIVmac239) present in the plasma following depletion of CD8ɑ+ cells (Fig. 3 and Fig. S2), this protection was highly variable, with one viremic animal exhibiting a viral population consisting of nearly 100% rechallenge virus following rechallenge and one aviremic animal containing only a single time point where the rechallenge virus was detectable post-depletion. While the proportion of rechallenge virus was low in this animal (Fig. 3B), it highlights that the rechallenge virus was able to establish a persistent, low-level infection despite immune control of plasma viremia, and that this rechallenge virus could be controlled spontaneously. While there is a possibility that the rechallenge virus did indeed seed the viral reservoir but was unable to replicate to detectable levels, we were unable to examine the sequences of proviral DNA isolated from CD4+ T cells in these animals due to the low number of SIV DNA per million PBMC (reported in Harwood et al. [33]). However, we feel this is unlikely when considering that transient bursts of replication in viremic animals were unable to significantly “reseed” the reservoir and alter the likelihood of that lineage being detected post-depletion (Table 1). Furthermore, a previous study conducted by our group showed that MCMs who were vaccinated with an attenuated SIVmac239∆nef and subsequently challenged with SIVmac239 found that only one of seven animals showed evidence of superinfection, even when the animals were given a CD8β-depleting antibody (63), supporting our conclusion that the possibility of undetected superinfection in these animals is unlikely. Future studies examining differences in susceptibility to reinfection, particularly in PTCs, may have important implications in our understanding of reservoir dynamics.

This study does have certain limitations. Of note, the viremic and aviremic animals were part of a previous therapeutic vaccine study; however, no differences in viral kinetics were observed during the 2-month period of follow-up (32). Interestingly, the CD8+ T cells in animals that received the vaccine regimen (closed shapes) appeared to reappear in the plasma more rapidly following CD8ɑ+ cell depletion than animals that did not receive the vaccine regimen (open shapes) (Fig. S4A). Unfortunately, due to the heterogeneity observed in these animals, we are unable to draw conclusions regarding the relationship between the rate at which CD8ɑ+ cells repopulate the blood and the composition of the rebounding viral population. Finally, the high observed rates of PTC in MCM stand in contrast to human cohorts, potentially reflecting a combination of host-specific immune responses and the control of experimental parameters such as infectious dose and ART initiation. This distinction highlights the importance of using caution when translating results to both human and rhesus macaque studies. While we focused on the CD8ɑ+ cell response to SIV, additional immunologic factors are likely involved in the establishment and maintenance of PTC, such as increases in antiviral monocytes (64), induction of protective type I interferon responses by innate immune cells (65–67), and the development of neutralizing antibodies (35, 37), and warrant further investigation.

Cumulatively, this study suggests a unique predisposition for MCM to establish PTC, highlights the importance of early ART initiation in restricting viral diversity and enhancing the likelihood of PTC, and emphasizes the importance of antiviral CD8+ cells in controlling viral replication during PTC. Further investigation into this potential model may allow us to explore the contribution of antiviral CD8+ cells to PTC. We provide evidence that while the composition of the viral reservoir remained stable during ART treatment and periods of PTC, small bursts of transient viral replication may influence this composition, even if viremia is spontaneously re-controlled. This finding highlights the importance of understanding mechanisms supporting viral reservoir maintenance in the absence of ongoing detectable viral replication. Furthermore, we found that animals with a longer duration of SIV infection prior to ART initiation had more diverse viral populations pre- and post-ART, as well as detectable viremia substantially earlier than their Early ART counterparts (Fig. 6 and 7). In sum, the data presented here provide evidence for a nonhuman primate model of PTC, which may allow researchers to more comprehensively understand the contribution of antiviral CD8+ cells in establishing and maintaining control of viremia in the absence of continual ART, as well as how these cells may act to restrict viral evolution during periods of PTC.

## MATERIALS AND METHODS

### Animal care and use statement

All samples used in this study were collected from a previous study (32, 33). A list of animal IDs, challenge virus, MHC genotype as determined by Wiseman et al. (68), and cohort is provided in Table S1. All animals were male between the ages of 6.4 and 12 years. All macaques were cared for by the staff at the Wisconsin National Primate Research Center (WNPRC) in accordance with the regulations, guidelines, and recommendations outlined in the Animal Welfare Act, the Guide for the Care and Use of Laboratory Animals, and the Weatherall Report.

### SIVmac239M infection and SIVmac239 rechallenge

The animal study was originally described in Harwood et al. (32, 33). For the Early ART cohort, eight MCMs positive for at least one copy of the M3 haplotype were infected intravenously with 10,000 infectious units (IU) of SIVmac239M suspended in 1 mL phosphate-buffered saline (PBS). Sixteen months following SIVmac239M infection, all animals were rechallenged intravenously with 100 TCID_50_ SIVmac239 suspended in 0.5 mL. For the Late ART cohort, five MCMs were infected intrarectally with 3,000 TCID50 of SIVmac239.

### CD8ɑ+ cell depletion

Eight SIVmac239M-infected MCMs (Table S1) were depleted of CD8ɑ+ cells as described in Harwood et al. (32, 33). Briefly, animals were given a single intravenous infusion of 50 mg/kg CD8ɑ-depleting antibody (MT807R1). The rhesus macaque IgG1 recombinant Anti-CD8α (MT807R1) monoclonal antibody was engineered and produced by the Nonhuman Primate Reagent Resource (NIH Nonhuman Primate Reagent Resource Cat# PR-0817, RRID:AB_2716320). Depletion of CD8ɑ+ T cells and NK cells was confirmed using a flow cytometry panel (33).

### SIV *gag* viral load quantification

SIV viral loads were quantified using a *gag* qPCR assay as previously described (69). Briefly, viral RNA was isolated from plasma using a Maxwell Viral Total Nucleic Acid kit (Promega), reverse transcribed, and amplified with the SuperScript III Platinum one-step quantitative RT-PCR system (Thermo Fisher Scientific). Viral RNA was quantified by quantitative PCR (qPCR) analysis on a LightCycler480 (Roche) and compared to an internal standard curve on each run. The assay used has a detection limit of 100 *gag* copy equivalents (ceq) per mL of plasma. The limit of detection (100 SIV *gag* ceq/mL) was reported when the viral load was at or below the limit of detection.

### SIV barcode sequencing

SIV barcode sequencing was performed as previously described (24) for all samples with a viral load of at least 1,000 viral copies/mL of plasma or lymphoid tissue. Briefly, samples were reverse transcribed using SuperScript III (Thermo Fisher Scientific) and a primer specific for a region adjacent to the barcode (Vpr.cDNA: 5′-CAG GTT GGC CGA TTC TGG AGT GGA TGC-3′ at position 6406–6380) per the manufacturer’s instructions. Cycling conditions were as follows: 50°C for 1 h; 55°C for 1 h; 70°C for 15 min; 10°C hold. 2U of RNAse H (Thermo Fisher Scientific) was then added to the reaction, and samples were incubated at 37°C for 20 min. Following cDNA synthesis, samples were PCR amplified using High Fidelity Platinum Taq (Thermo Fisher Scientific) per the manufacturer’s instructions and custom primers containing the Illumina index and adapter sequences (Table S2). PCR cycling conditions were as follows: 94°C for 2 min; 40× (94°C for 15 s, 60°C for 90 s, 68°C for 30 s); 68°C for 5 min; 10°C hold. Following PCR amplification, samples were subjected to a 1.2:1 AMPure bead clean-up step, then quantified using a Qubit High-Sensitivity quantification assay (Thermo Fisher Scientific), and amplicon lengths were determined using an Agilent BioAnalyzer (Agilent). Samples were then pooled equimolarly and sequenced on a 2 × 150 Illumina MiSeq.

### SIVmac239M barcode analysis

SIV samples were sequenced from plasma that had a minimum viral load of 1,000 copies/mL of plasma, corresponding to approximately 60 vRNA input templates. A summary of total sequences per primer, total sequences matching known barcodes, sample sequencing depth, total barcodes per sample, and sequencing input is included in Table S3. SIV barcode sequences were identified from the R1 read using a tool developed in the Keele lab and available at https://github.com/KeeleLab. All samples were sequenced in duplicate, and samples with less than 5,000 reads were excluded. For each time point, barcode counts (the number of times a given barcode was identified in that sample) were pooled. SIVmac239M barcodes were included in the analysis if they were present in the pre-ART peak viral load and were above the minimum input threshold of 1/minimum number of input templates. Barcode sequences with a hamming distance of 1 from a known barcode were included in the analysis if they met the above criteria; otherwise, barcodes containing multiple mismatches were discarded as sequencing artifacts. Lineage-specific viral loads depicted in Fig. S2 were estimated by identifying the relative proportion a lineage comprised in the total viral population and multiplying it by the total viral load. As such, lineage-specific values may fall below the assay limit of detection, representing proportional estimates rather than independently measured or quantified viral loads. Logistic regressions were calculated using the glm function in R. For the single model evaluating post-ART rebound (Table 1, Model 1), the pre-ART viral load AUC was the sole explanatory variable. For Model 2, pre-ART viral load AUC was used as the sole explanatory variable. The next two models included the pre-ART viral load AUC and a binary indicator variable (detection of the lineage post-ART or post-rechallenge) as separate covariates. The R package DescTools was used to calculate viral load area under the curve (AUC).

### Whole-genome sequencing

SIV whole-genome sequencing was performed as previously described for samples with at least 1,000 viral copies/mL of plasma (70). Briefly, vRNA was isolated from plasma using the Maxwell Viral Total Nucleic Acid kit (Promega). Four overlapping amplicons (A, B, C, and D) spanning the entire SIV coding sequence were generated for each sample using a SuperScript III one-step reverse transcription (RT)-PCR system with high-fidelity Platinum Taq (Invitrogen) and the primers listed in Table S4 with the following PCR cycling conditions: 50°C for 60 min; 94°C for 2 min; 2× (94°C for 15 s, 60°C for 1 min, 68°C for 4 min); 2× (94°C for 15 s, 58°C for 1 min, 68°C for 4 min); 41× (94°C for 15 s, 55°C for 1 min, 68°C for 4 min); 68°C for 10 min; 10°C hold. Following PCR amplification, samples were subjected to a 1:1 AMPure bead clean-up step, then quantified using a Qubit High-Sensitivity quantification assay (Thermo Fisher Scientific). The amplicons for all samples were pooled equimolarly and used to generate uniquely tagged libraries using a Nextera XT kit (Illumina), per the manufacturer’s instructions. Samples were then sequenced in-house on a 2 × 250 Illumina MiSeq at 10 pM with a 1% PhiX spike.

In the case of samples with amplicon dropouts and low viral loads, samples were amplified using an SIV multiplex PCR protocol as previously described (71). Briefly, cDNA was generated from vRNA using SuperScript IV (Invitrogen). Samples were then amplified using the Q5 Polymerase (New England BioLabs) and the following PCR cycle conditions: 98°C for 30 s, 35× (95°C for 15 s and 65°C for 5 min); 4°C hold. Amplified products were quantified using a Qubit dsDNA High-Sensitivity assay (Thermo Fisher) and prepared for sequencing using an Illumina TruSeq kit per the manufacturer’s instructions. Prepared samples were pooled equimolarly and sequenced in-house on a 2 × 250 Illumina MiSeq at 10 pM with a 10% PhiX spike.

### Epitope identification and analysis

Following sequencing and demultiplexing, paired-end reads were merged using bbtools (72). Merged fastq files were mapped to the SIVmac239M reference and trimmed based on a minimum quality score. The epitope sequences for each animal and time point were identified, translated, and counted by amino acid sequence using a computational pipeline available on our GitHub (https://github.com/rvmoriarty/PTC_MCM). The Bray-Curtis dissimilarity index was calculated using the vegan R package (73). Statistical analysis was done using Prism 9 and R. Shapiro-Wilk tests were used to determine normality. For normally distributed data, t-tests were performed. For non-normally distributed data, Mann-Whitney U tests were performed.

### Flow cytometry

Flow cytometry was used to determine the amount and phenotype of CD8+ T cells present in the PBMC following CD8ɑ+ cell depletion as previously described; gating schematics and lymphocyte population identification are also described (32, 33). Briefly, previously cryopreserved PBMCs isolated from whole blood were used to assess the frequency of T-cell populations longitudinally. Cells were thawed, washed with RPMI + 10% FBS (R10), and rested for 30 min at room temperature in a buffer consisting of 2% FBS in 1× PBS (2% FACS buffer) with 50 nM dasatinib (Thermo Fisher Scientific). Cells were then washed with 2% FACS buffer with 50 nM dasatinib and incubated at room temperature with Gag_386–394_GW9 and Nef_103–111_RM9 tetramers for 45 min. Cells were washed with 2% FACS buffer with 50 nM dasatinib and incubated with the remaining surface markers for 20 min at room temperature. Cells were then washed with 2% FACS buffer, fixed for a minimum of 20 min with 2% paraformaldehyde, and acquired immediately using a FACS Symphony A3 (BD Biosciences). The data were analyzed with FlowJo software for Macintosh (BD Biosciences, version 10.8.0). Cell subpopulations were excluded from analysis when the parent population contained <50 events. Antibodies used are listed in Table 2 . Tetramers were generated as previously described (32, 33).

**TABLE 2:** Antibodies used to determine CD8+ cell frequencies in PBMC

Antibody	Clone	Fluorochrome
Live/dead	–^*a*^	Near-infrared
CD3	SP34-2	AF700
CD4	SK3	BUV737
CD8	DK25	APC
Gag_386–394_GW9 tetramer	–	PE
Nef_103–111_RM9 tetramer	–	BV421
NKG2A	Z199	PE-Cy7

^*a*^ –, not applicable.

## Data Availability

All whole-genome viral sequences used are available on the Sequence Read Archive (SRA) under accession number PRJNA1463161. All scripts used to generate and organize data are available upon reasonable request or on our GitHub (https://github.com/rvmoriarty/PTC_MCM). SIV barcodes were identified using a computational pipeline written in R, which can be downloaded from https://github.com/KeeleLab.

## SUPPLEMENTAL MATERIAL

Figure S1 jvi.00041-26-s0001.tif Cellular responses to SIV antigens during PTC.

Figure S2 jvi.00041-26-s0002.tiff Log_10_ viral copies for each SIVmac239M lineage throughout the course of the study in viremic and aviremic animals.

Figure S3 jvi.00041-26-s0003.tiff Proportion of rechallenge virus detected in the plasma immediately following intravenous infusion of SIVmac239.

Figure S4 jvi.00041-26-s0004.tiff Frequencies of bulk CD8+ T cells, NK cells, CD8+Gag GW9 tetramer+ cells, and CD8+Nef RM9 tetramer+ cells post-CD8ɑ+ cell depletion.

Figure S5 jvi.00041-26-s0005.tiff Bray-Curtis dissimilarity index for Gag GW9, Nef RM9, and Rev SP10 epitope sequences pre- and post-ART.

Table S1 jvi.00041-26-s0006.tiff Animal IDs, challenge virus, MHC genotype, age, sex, and cohort for all animals used in this study.

Table S2 jvi.00041-26-s0007.tiff Primer sequences used in sequencing the SIVmac239M barcode.

Table S3 jvi.00041-26-s0008.xlsx Summary data of SIV barcode sequencing results as determined by the R script provided on the Keele Lab Github.

Table S4 jvi.00041-26-s0009.tiff Primer names and sequences used for whole-genome sequencing using the four-amplicon approach.

Table S5 and Legends jvi.00041-26-s0010.docx Proportion of variant epitope sequences present in stock SIVmac239 and SIVmac239M viruses used for infection; legends for all supplemental material.

ASM does not own the copyrights to Supplemental Material that may be linked to, or accessed through, an article. The authors have granted ASM a non-exclusive, world-wide license to publish the Supplemental Material files. Please contact the corresponding author directly for reuse.
