# Supplementary figures and images for: CD8ɑ+ cells suppress SIV replication without the development of mutations within MHC class-I-restricted epitopes during post-treatment control
Source: J Virol. 2026 Jun 15;100(7):e00041-26. doi: 10.1128/jvi.00041-26 (PMC13386877; doi:10.1128/jvi.00041-26)

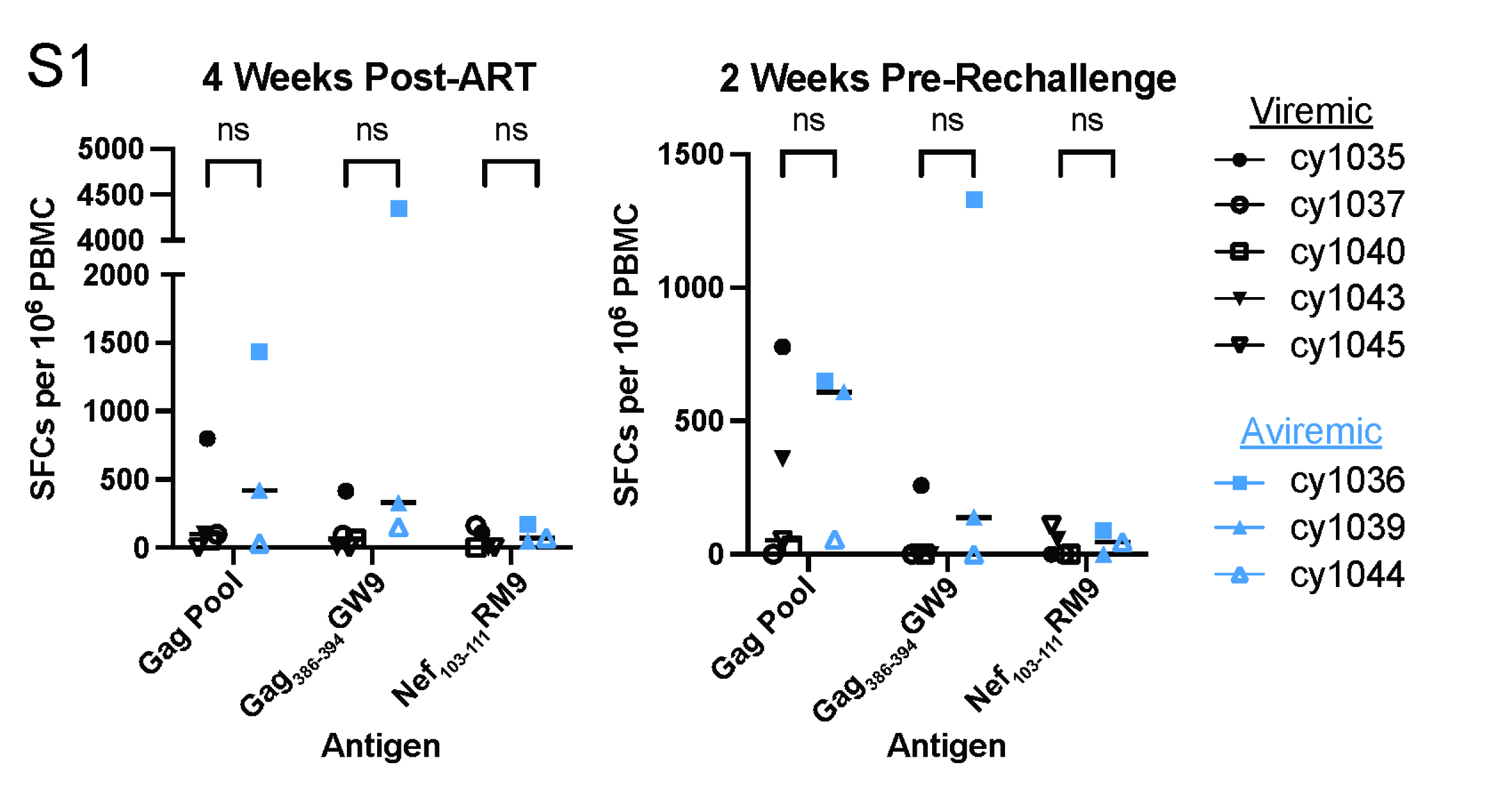

Supplement: Figure S1 — Cellular responses to SIV antigens during PTC. [file jvi.00041-26-s0001.tif]

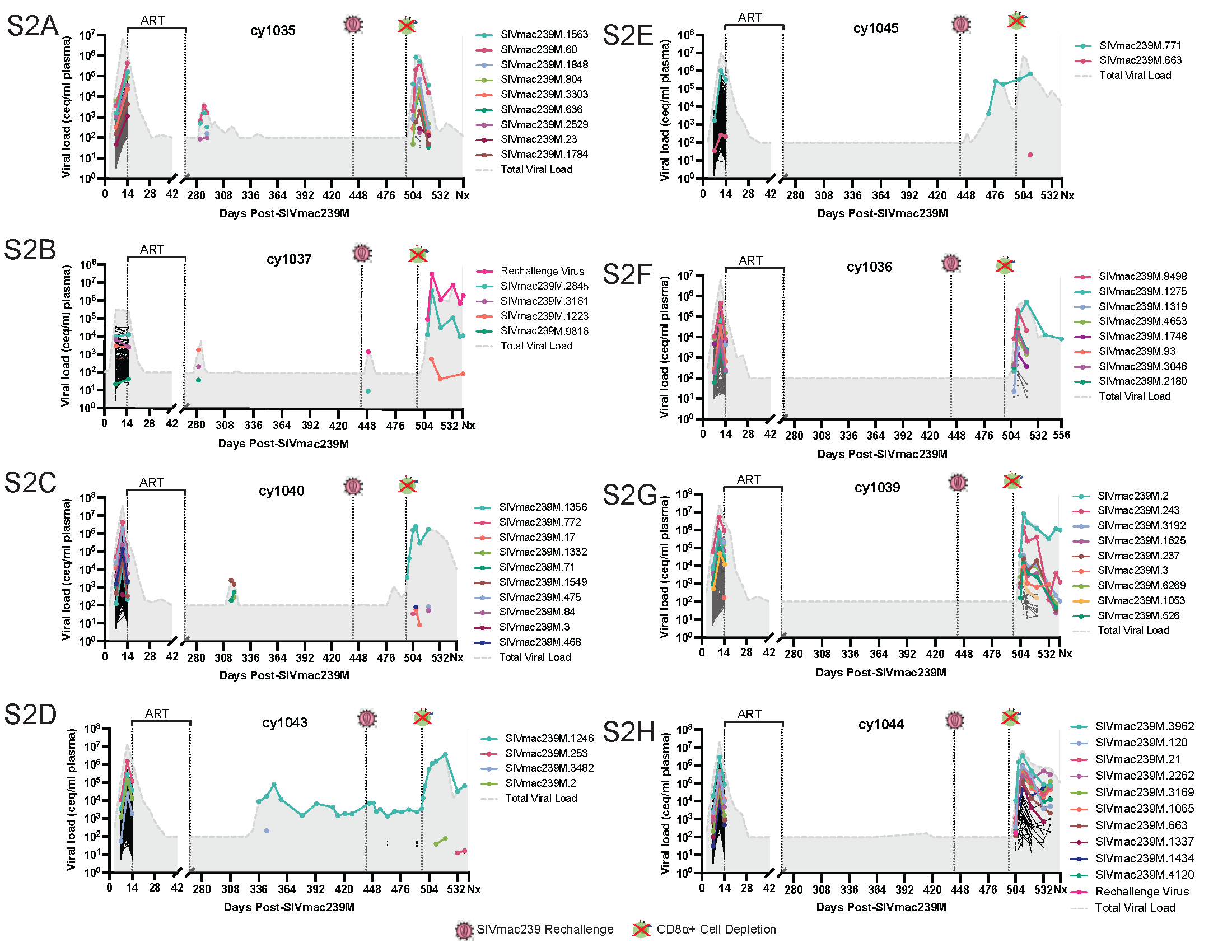

Supplement: Figure S2 — Log10 viral copies for each SIVmac239M lineage throughout the course of the study in viremic and aviremic animals. [file jvi.00041-26-s0002.tiff]

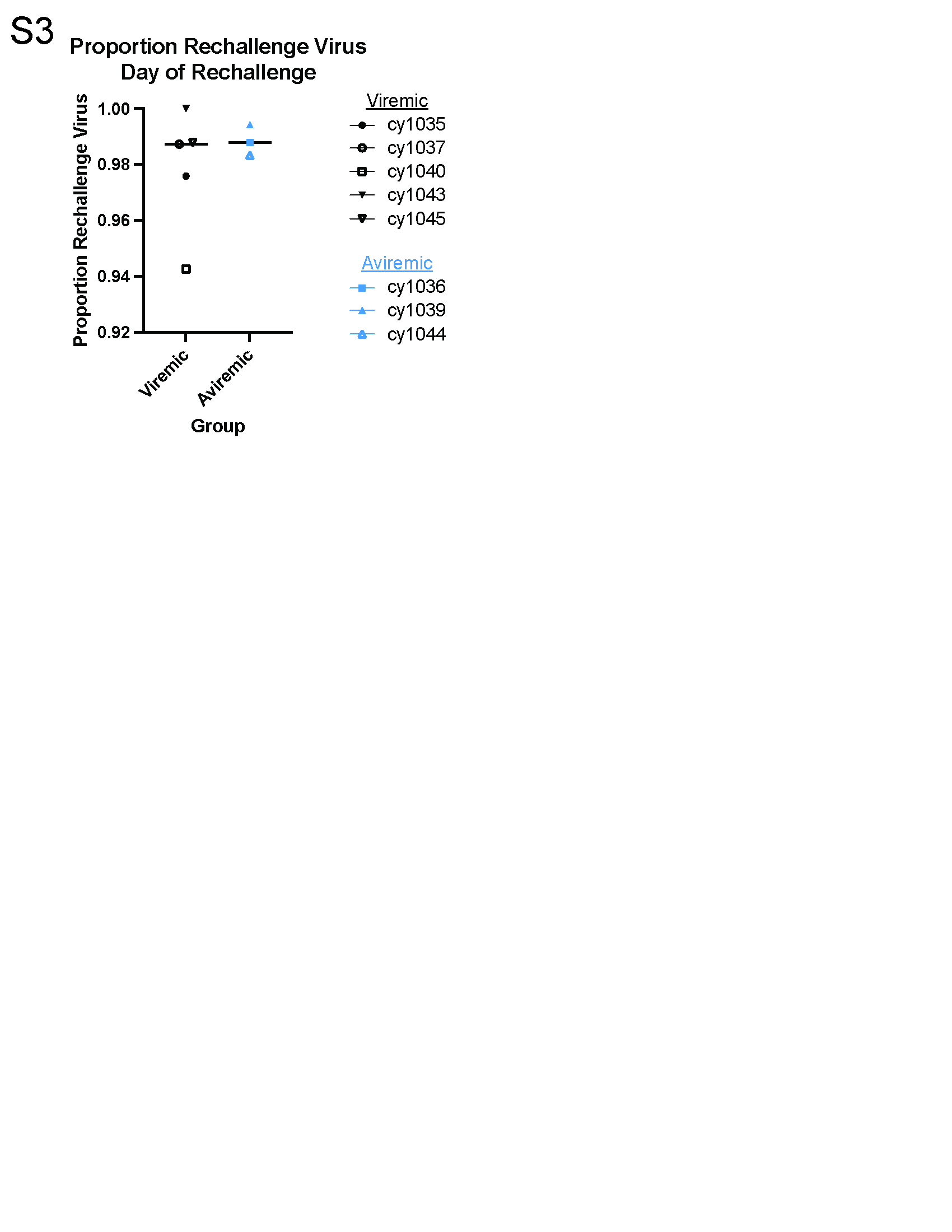

Supplement: Figure S3 — Proportion of rechallenge virus detected in the plasma immediately following intravenous infusion of SIVmac239. [file jvi.00041-26-s0003.tiff]

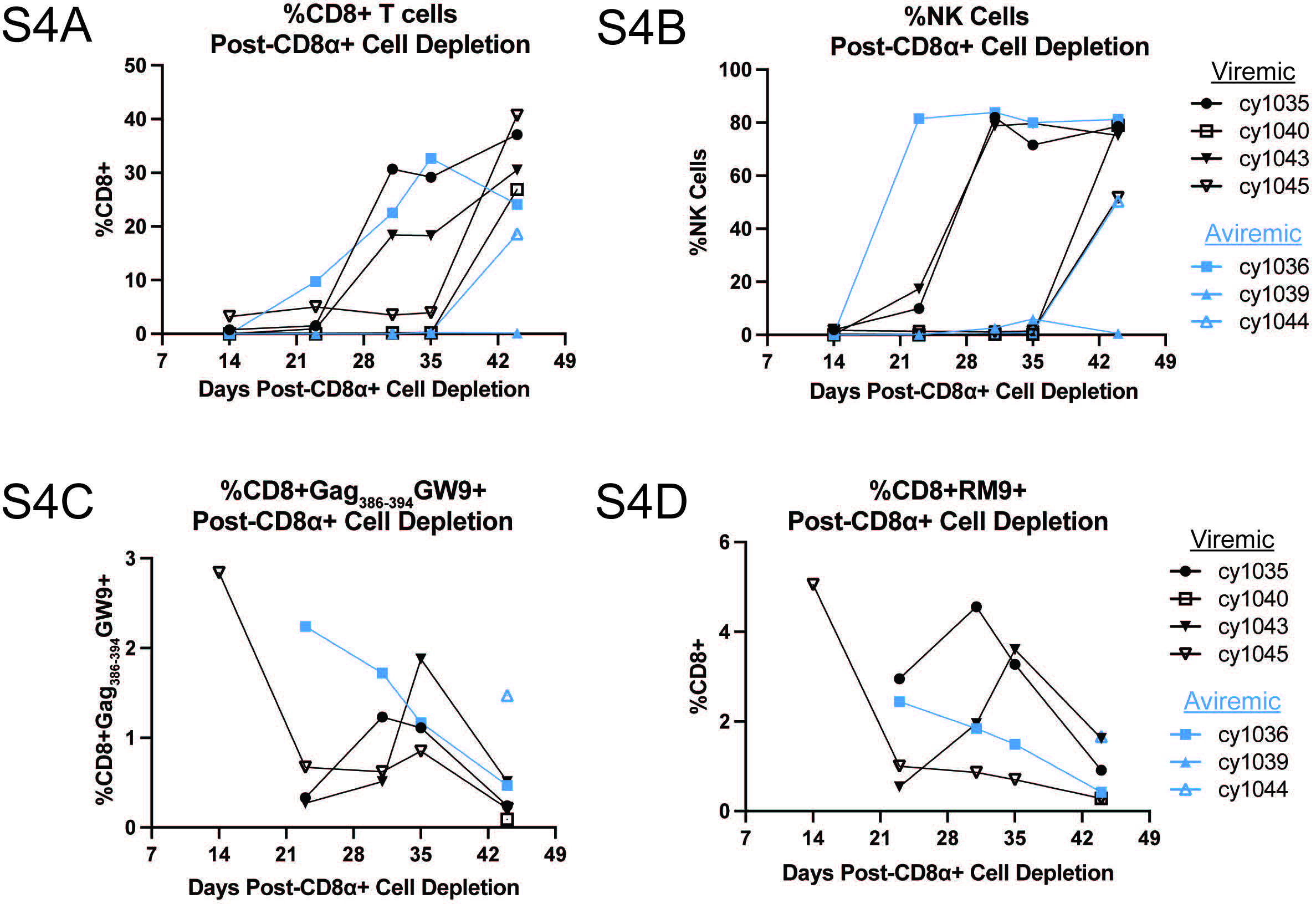

Supplement: Figure S4 — Frequencies of bulk CD8+ T cells, NK cells, CD8+Gag GW9 tetramer+ cells, and CD8+Nef RM9 tetramer+ cells post-CD8ɑ+ cell depletion. [file jvi.00041-26-s0004.tiff]

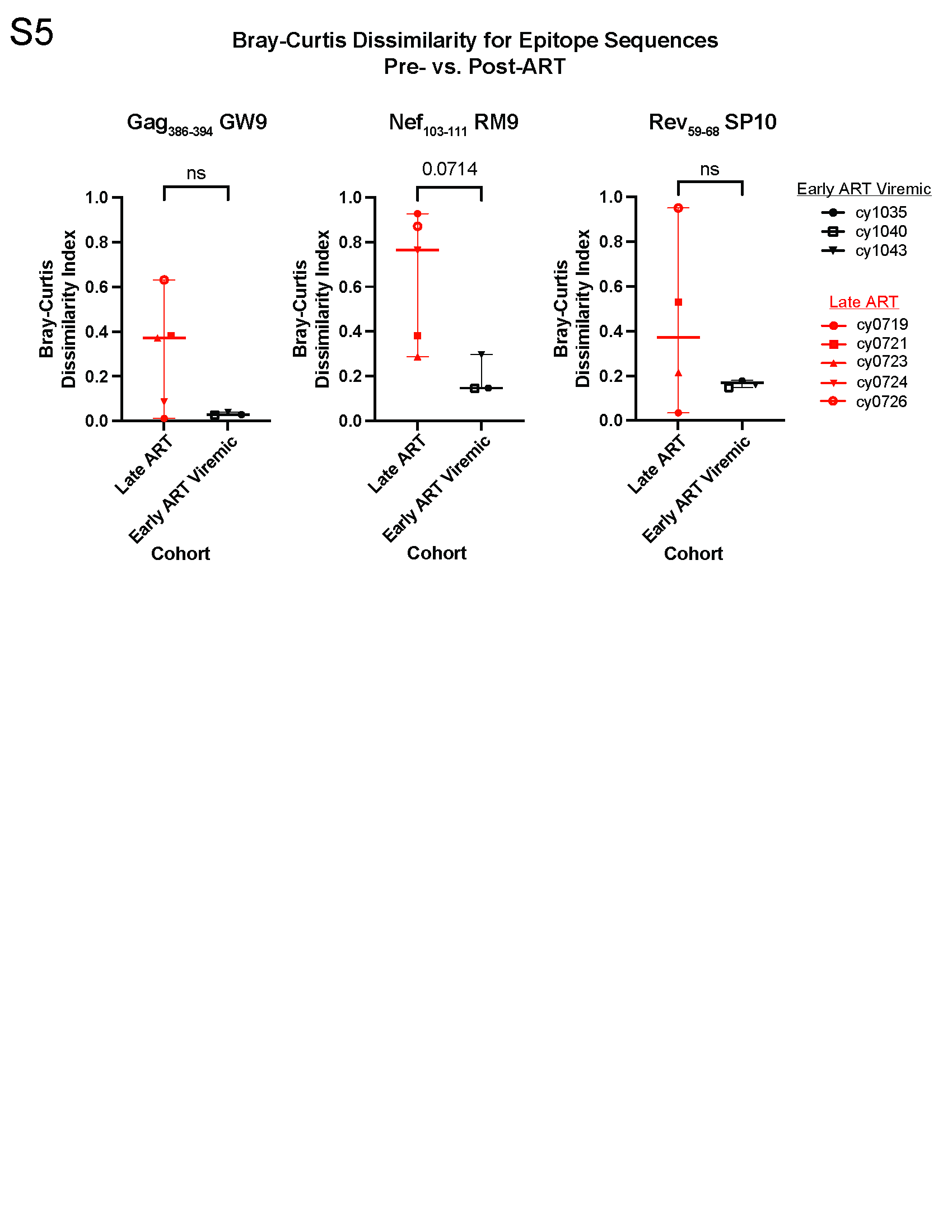

Supplement: Figure S5 — Bray-Curtis dissimilarity index for Gag GW9, Nef RM9, and Rev SP10 epitope sequences pre- and post-ART. [file jvi.00041-26-s0005.tiff]

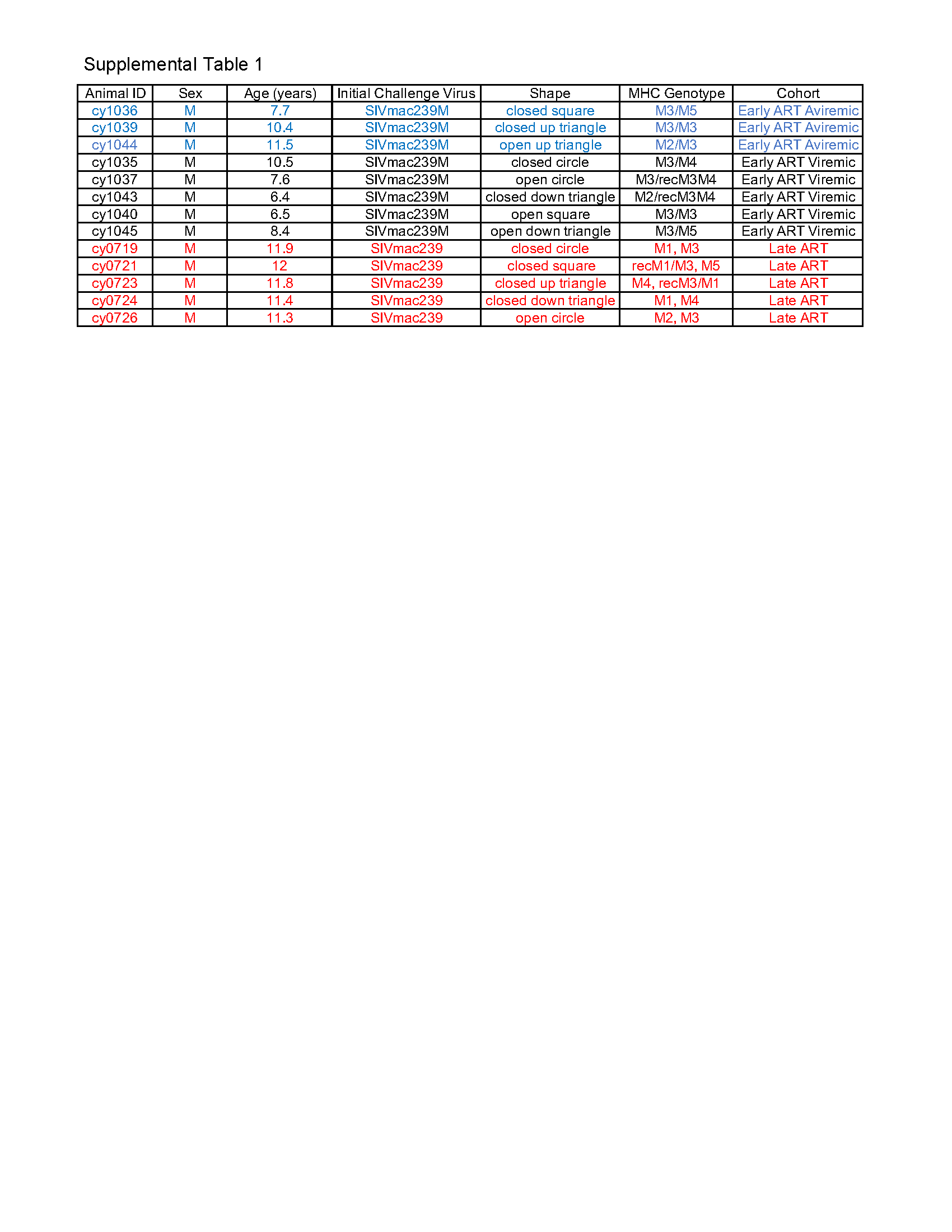

Supplement: Table S1 — Animal IDs, challenge virus, MHC genotype, age, sex, and cohort for all animals used in this study. [file jvi.00041-26-s0006.tiff]
