# Supplementary material for: CD8ɑ+ cells suppress SIV replication without the development of mutations within MHC class-I-restricted epitopes during post-treatment control
Source: J Virol. 2026 Jun 15;100(7):e00041-26. doi: 10.1128/jvi.00041-26 (PMC13386877; doi:10.1128/jvi.00041-26)
Supplement: Table S5 and Legends — Proportion of variant epitope sequences present in stock SIVmac239 and SIVmac239M viruses used for infection; legends for all supplemental material. [file jvi.00041-26-s0010.docx]

Supplemental Table 5:

| Epitope | Sequence | SIVmac239 Stock Variant Proportion | SIVmac239M Stock Variant Proportion |
| --- | --- | --- | --- |
| Gag GW9 | ....S.... | 0.00020644 | 0.00064772 |
|  | ....T.... | 0.00010322 | 0.0002429 |
|  | .S....... | 0.000382 | 0.00032386 |
|  | ..K...... | 0.00010322 | 0.00008097 |
|  | .S.R..... | 0 | 0 |
|  | ..I...... | 0 | 0.00040483 |
|  | GPRKPIKCW (WT) | 0.98472337 | 0.97069063 |
| Nef RM9 | .SR...... | 0 | 0 |
|  | ..G...... | 0 | 0 |
|  | ..R.S.... | 0 | 0 |
|  | .S....... | 0.00046083 | 0.00115574 |
|  | .TR...... | 0 | 0 |
|  | K.R...... | 0 | 0 |
|  | ....S.... | 0.00046083 | 0.00028893 |
|  | ....L.... | 0.00046083 | 0.00028893 |
|  | .L....... | 0.00046083 | 0.0004334 |
|  | ..R...... | 0.00046083 | 0.0004334 |
|  | .......P. | 0 | 0 |
|  | ..N...... | 0.00046083 | 0 |
|  | K........ | 0.00046083 | 0.00057787 |
|  | ..Q...... | 0 | 0 |
|  | ...A..... | 0.00092166 | 0.0013002 |
|  | RPKVPLRTM (WT) | 0.97963017 | 0.9875576 |
| Rev SP10 | ..N...... | 0 | 0 |
|  | .....S.... | 0.00016969 | 0.00021615 |
|  | ...A...... | 0 | 0.00054037 |
|  | .I........ | 0.00016969 | 0.00010807 |
|  | .........S | 0.00016969 | 0.00021615 |
|  | .........H | 0.00016969 | 0.00021615 |
|  | .........T | 0.00033939 | 0.00010807 |
|  | ...G...... | 0.00101816 | 0.00064844 |
|  | SFPDPPTDTP (WT) | 0.98184286 | 0.980763 |

Supplemental Table 1. Animal IDs, challenge virus, MHC genotype, age, sex, and cohort for all animals used in this study. Animals are color coded by cohort. MHC genotypes were determined as previously described (65).

Supplemental Table 2. Primer sequences used in sequencing the SIVmac239M barcode.

Supplemental Table 3. Summary data of SIV barcode sequencing results as determined by the R script provided on the Keele Lab Github. Each row contains a single sample sequenced. Run Number refers to the sequencing run each sample was on. Date references the date the sample was collected. Barcodes refers to the primer used for indexing, with primer sequences provided in Supplemental Table 2. Number of 5’ Indexing Sequences indicates the total number of reads that were extracted matching the barcode primer used. Total Number of Sequences Extracted per Primer was determined by the number of sequences extracted by a given primer that also matched the reference. Number of Sequences Matching Known Barcode was determined by calculating which extracted sequences also contained a barcode in the barcode reference file found on the Keele Lab GitHub. Number of Known Barcodes indicates the number of unique barcodes found in a given sample that are also present in the barcode reference file. Sequencing Input references the estimated number of input templates sequenced.

Supplemental Table 4. Primer names and sequences used for whole-genome sequencing using the four-amplicon approach, described in Sutton et al (67).

Supplemental Table 5. Baseline sequence diversity within MHC-I-restricted CD8+ T cell epitopes in the SIVmac239 and SIVmac239M challenge stocks. Relative abundance of the wild-type (WT) and variant epitope sequence relative to the reference sequence (accession M33262). Dots indicate amino acids matching the WT sequence with letters indicating the variant amino acid.

Supplemental Figure 1. Cellular responses to SIV antigens during PTC. IFNγ ELISPOT assays were performed four weeks following ART interruption (left), two weeks prior to isogenic rechallenge (right), an one week following isogenic rechallenge (bottom) to examine responses to a pool of SIV Gag peptides, Gag_386-394_GW9, and Nef_103-111_RM9 (data recategorized from Harwood et al (32, 33)) . Lines represent medians. Symbols indicate animal and group, with closed symbols indicating animals that were vaccinated as part of a previous study, described in the results section. Statistical significance was determined using a Mann-Whitney U test.

Supplemental Figure 2. Log_10_ viral copies (Y axis) for each SIVmac239M lineage throughout the course of the study in viremic (A-E) and aviremic (F-H) animals. Each lineage is indicated by a different color, with only the top 10 lineages detected post-depletion colored due to color and space constraints. The remaining lineages are shown as grey lines and circles. Rechallenge virus is indicated in pink. Total log_10_ viral load is shown in the grey shaded area. Lineage-specific values were estimated by multiplying relative abundance in the total viral population by the measured total viral load. Values below the assay limit of detection should be interpreted as relative estimates of lineage contribution.

Supplemental Figure 3. Proportion of rechallenge virus detected in the plasma immediately following intravenous infusion of SIVmac239. Each animal is indicated by a unique color and shape combination, with viremic animals shown in black and aviremic animals shown in blue. Line represents median of each group.

Supplemental Figure 4. Frequency of bulk (A), Gag_386-398_GW9 tetramer+ (B), and Nef_103-111_RM9 tetramer+ (C) CD8+ T cells post-CD8ɑ+ cell depletion. Animals are coded by unique symbol and color combinations, with viremic animals in black and aviremic animals in blue. For subpopulation analysis, only samples in which the parent population of CD8+ cells had at least 50 events were included.

Supplemental Figure 5. Bray-Curtis dissimilarity index for Gag_386-398_GW9 (left), Nef_103-111_RM9 (center), and Rev_59-68_SP10 (right) epitope sequences pre- and post-ART. Early ART viremic animals are in unique black symbols and Late ART animals are in unique red symbols. Significance was determined using Mann-Whitney U tests.
